# Gadolinium-Doped Iron Oxide Nanoparticles Enhance Radiosensitivity in Melanoma Models Associated with Metabolic Dysfunction

**DOI:** 10.3390/pharmaceutics18050525

**Published:** 2026-04-26

**Authors:** Roxana Cristina Popescu, Cosmin Catalin Mustaciosu, Adrian-Ionut Nicoara, Paul Emil Mereuta, Verena Kopatz, Roxana Trusca, Stela Patrascu, Elif Menlivuap, Cosmin-Florin Sovan, Diana Iulia Savu, Sorin Ion Jinga

**Affiliations:** 1 Department of Bioengineering and Biotechnology, Faculty of Medical Engineering, National University of Science and Technology Politehnica Bucharest, Gheorghe Polizu Str. 1-7, RO-011061 Bucharest, Romania; elif.tn02@gmail.com (E.M.); cosmin_florin.sovan@stud.fim.upb.ro (C.-F.S.);; 2 Department of Life and Environmental Physics, National Institute for Research and Development in Physics and Engineering “Horia Hulubei”, Reactorului Str. 30, RO-077125 Magurele, Romania; dsavu@nipne.ro; 3 Department of Science and Engineering of Oxide Materials and Nanomaterials, Faculty of Chemical Engineering and Biotechnologies, National University of Science and Technology Politehnica Bucharest, Gheorghe Polizu Str. 1-7, RO-011061 Bucharest, Romania; 4 National Research Center for Micro and Nanomaterials, National University of Science and Technology Politehnica Bucharest, Splaiul Independentei 313, RO-060042 Bucharest, Romania; 5 Department of Applied Nuclear Physics, National Institute for Research and Development in Physics and Engineering “Horia Hulubei”, Reactorului Str. 30, RO-077125 Magurele, Romania; 6 Department of Radiation Oncology, Medical University of Vienna, Waehringer Guertel Str. 18-20, 1090 Vienna, Austria; 7 Department of Radioisotopes and Radiation Metrology, National Institute for Research and Development in Physics and Engineering “Horia Hulubei”, Reactorului Str. 30, RO-077125 Magurele, Romania

**Keywords:** gadolinium-doped iron oxide nanoparticles, radiosensitization, melanoma, 3D tumor spheroids, metabolic dysfunction, preclinical radiotherapy

## Abstract

**Background.** Nanoparticle-mediated radiotherapy is a promising approach to enhance tumor radiosensitivity while reducing damage to healthy tissues. Particularly, melanoma is a highly aggressive malignancy with an increasing global incidence and limited therapeutic options in advanced stages, due to its intrinsic radioresistance and narrow therapeutic window in metastatic settings. In this study, we developed a systematic library of gadolinium-doped iron oxide nanoparticles (Fe-Gd NPs) with controlled compositions (0–75% Gd) to investigate the functional and compositional determinants of radiosensitization in melanoma. **Methods.** The physicochemical properties of the Fe-Gd NPs, including the morphology, crystallinity, and composition, were thoroughly characterized and correlated with biological responses. The biological evaluation was performed using both 2D and tissue-relevant 3D melanoma models, integrating metabolic viability assays (MTT/MTS), mitochondrial function (ATP quantification, MitoTracker analysis), and clonogenic survival following low-energy X-Ray irradiation (150 kV, 4 Gy). In vivo systemic tolerance and response in non-tumor tissues were investigated in BALB/C mice. **Results.** Our results showed that radiosensitization did not increase linearly with the Gd content, with the 25% Fe-Gd NPs being identified as a therapeutic window and having the most pronounced effect in melanoma cell models, while maintaining good systemic safety in vivo. This study provides functional evidence that nanoparticle-mediated radiosensitization is not only determined by a high Z content, but also by tumor-specific metabolic adaptability and the nanoparticle composition. **Conclusions.** These findings support the rational design of Fe-Gd nanoparticles with optimized therapeutic windows and highlight the importance of metabolic and 3D tissue-relevant models in preclinical evaluation of nanoparticle-mediated radiotherapy.

## 1. Introduction

Although melanoma is a cutaneous cancer that is visible at skin level, it continues to represent a complex public health problem, as incidence rates are steadily increasing worldwide. According to the most recent global estimates from 2022, nearly 332,000 new melanoma cases and approximately 59,000 deaths were reported, with mortality being largely associated with advanced disease stages and a high incidence of metastasis, particularly to distant organs such as the brain [1]. Due to its disproportionately high mortality relative to incidence, together with intrinsic or acquired resistance to standard therapeutic approaches, including radio- and chemotherapy, melanoma remains a major therapeutic challenge in advanced stages [2,3], underscoring the need for the development of new treatment strategies [4].

Melanoma is generally considered as relatively radio-resistant tumor, requiring high radiation doses and specific fractionation schemes to achieve therapeutic efficacy [5,6,7,8]. Moreover, metastatic sites such as the brain are particularly susceptible to radiation-induced toxicity, resulting in a narrow therapeutic window [9,10,11]. In this context, nanoparticle-mediated radiotherapy has emerged as a promising oncological strategy, aiming to enhance the local radiation dose through the generation of secondary radiation and reactive oxygen species upon exposure to ionizing radiation [12]. Due to their small dimensions, comparable to those of biological molecules, nanoparticles are capable of crossing biological barriers and targeting tumor tissue compartments [13,14]. However, despite their potential to overcome some of the limitations of conventional radiotherapy, the efficiency of nanoparticle-mediated radiotherapy clinically depends on the nanoparticle composition, physicochemical properties, mechanism of action, and resulting biological response [15,16,17].

Iron (Fe) oxide nanoparticles have been extensively investigated in a wide range of biomedical applications under clinically relevant conditions [18], including nanoparticle-mediated radiotherapy [19]. These materials have demonstrated high biocompatibility with living tissues [20] and efficient cellular internalization [21,22,23]. The biological performance of iron oxide nanoparticles is known to be highly dependent on their physicochemical properties, including their size, composition, and structural organization, which can significantly influence their interaction with cellular systems and therapeutic responses [24,25,26,27]. Numerous studies have reported the radiosenitizing potential of Fe oxide nanoparticles [28,29]; however, this effect remains constrained by the nanoparticle concentration, radiation dose, and radiation energy, largely due to the relatively low atomic number of Fe (Z_Fe_ = 26) [30,31,32,33].

In contrast, gadolinium (Gd) is a high-atomic-number element (Z_Gd_ = 64), exhibiting a strong potential for radiosensitization, demonstrated in both preclinical and clinical settings [34,35,36]. Gd-based materials are already widely used in clinical practice, primarily as contrast agents [37,38], and have shown efficacy in enhancing radiotherapeutic outcomes [36]. Nevertheless, the clinical translation of Gd-based nanoparticles is limited by the well-documented toxicity of free Gd^3+^ ions [39], necessitating stringent chemical and structural control. In this regard, the combination of Fe and Gd within a single nanoplatform offers the prospect of synergistic benefits, provided that the compositional window is carefully optimized and rigorously correlated with the resulting biological response.

In this regard, we report the synthesis of Gd-doped Fe oxide nanoparticles based on a previously developed Fe oxide formulation [22], which demonstrated high biocompatibility [22], as well as it’s radiosensitizing potential under low energy X-Ray irradiation in cervical adenocarcinoma models [32,33]. The rationale for incorporating Gd within the Fe oxide lattice was to enhance the antitumor efficiency while mitigating the known toxicity associated with free Gd^3+^ ions in biological environments.

In this study, a systematic series of Fe-Gd oxide nanoparticles with controlled Gd contents (0–75% Gd) was developed and thoroughly characterized, correlating key physicochemical parameters, such as morphology, composition, and crystallinity, with biological responses relevant to radiotherapy enhancement. The biological evaluation was performed in a stepwise manner, starting with conventional 2D cellular models and progressing toward tissue-relevant 3D multi-cellular melanoma spheroids, followed by the assessment of systemic tolerance in non-tumoral models.

To the best of our knowledge, this work represents one of the first comprehensive investigations that combines controlled Fe-Gd compositional tuning with an integrated functional biological evaluation, capable of functionally distinguishing metabolic viability, mitochondrial adaptation, and long-term clonogenic survival in the context of nanoparticle-mediated radiosensitization. Rather than assuming a linear relationship between the Gd content and therapeutic efficiency, the present approach allows the identification of composition-dependent effects and the existence of an optimal radiosensitizing window. Moreover, by integrating functional metabolic assays with structural evaluations in 3D tumor models, this study supports the concept that metabolic impairment can occur prior to detectable morphological alterations, thereby underscoring the limitations of relying exclusively on volume-based endpoints in preclinical radiotherapy assessments.

## 2. Materials and Methods

### 2.1. Synthesis of Nanoparticles

Iron- and/or gadolinium oxide- based nanoparticles conjugated with polyethylene glycol with a molecular weight of 6000 Da (PEG 6K) were synthesized using reagents of analytical grade obtained from Millipore Sigma GmbH (Darmstadt, Germany), including ferrous sulphate heptahydrate (FeSO_4_·7H_2_O), iron (III) chloride hexahydrate (FeCl_3_·6H_2_O), gadolinium (III) chloride hexahydrate (GdCl_3_·6H_2_O), 25% ammonia solution (NH_3_), polyethylene glycol 6000 Da, and 100% ethanol (EtOH).

Synthesis of Fe_3_O_4_ nanoparticles (0% Gd^3+^ Fe_3_O_4_@PEG 6K):

The precursor solution was prepared by dissolving 0.4 g of FeCl_3_ and 0.64 g of FeSO_4_ in 50 mL of deionized water ([Fe^3+^]:[Fe^2+^] = 0.625) [22]. The precipitation solution was obtained by mixing 8 mL of 25% NH_3_ with 400 mL of deionized water and was used for all nanoparticle preparations. The precursor solution was added dropwise to 100 mL of precipitation solution under magnetic stirring, resulting in the formation of a dark brown precipitate. The precipitate was washed three times with deionized water using magnetic separation and finally suspended in 200 mg of PEG 6K dissolved in 50 mL of EtOH. The suspension was stirred at 70 °C for 1 h to facilitate nanoparticle encapsulation within the polymer shell.

Synthesis of Gd_2_O_3_ nanoparticles (Gd_2_O_3_@PEG 6K):

The Gd precursor solution was prepared by dissolving 3 g of GdCl_3_ in 50 mL of deionized water. The solution was added dropwise to 100 mL of precipitation solution under magnetic stirring, yielding to a white precipitate. This precipitate was separated via low vacuum evaporation at 37 °C and washed three times with deionized water. The nanoparticles were then suspended in 200 mg of PEG 6K dissolved in 50 mL of EtOH and stirred at 70 °C for 1 h to ensure encapsulation.

Synthesis of Fe-Gd composite nanoparticles (10%/15%/25%/50%/75% Gd^3+^ @PEG 6K):

Composite Fe-Gd nanoparticles were obtained by partially substituting the Fe^3+^ precursor with the Gd^3+^ precursor, while maintaining a constant [A^3+^]:[B^3+^] ratio. Substitutions were performed at 10, 15, 25, and 50, respectively, and 75 wt%. After encapsulation, the nanoparticles were washed three times with deionized water and subsequently dispersed in deionized water via ultrasonication.

### 2.2. Physico-Chemical Characterization

#### 2.2.1. Scanning Electron Microscopy

The morphology of the synthesized nanoparticles was examined using scanning electron microscopy (SEM). The electron beam was accelerated at 30 kV and a probe current ranging from 1 pA to 300 μA. For SEM investigation, 100 μL of the nanoparticle suspension (2 mg/mL concentration) was deposited onto a Mylar foil, and the solvent was evaporated at 50 °C. Nanoparticle diameters were measured and statistically analyzed using ImageJ 1.54g software (National Institute of Health, Bethesda, MD, USA).

#### 2.2.2. X-Ray Diffraction

The crystallinity of the Fe and/or Gd oxide powders with varying compositions was investigated using X-Ray diffraction (XRD). All measurements were performed at room temperature on PANalytical Empyrean diffractometer equipment (Melvern-PANalytical, Almelo, The Netherlands), equipped with a copper X-Ray tube (λCuKα1 = 1.541874 Å). The samples were scanned in Bragg–Brentano geometry with a step size of 0.02° and a counting time of 100 s/step. Diffractograms were recorded over a 2θ range of 5–80°. Data analysis and identification of diffraction peaks were conducted using the X Pert High Score Plus 3.0 software (Melvern-PANalytical, Almelo, The Netherlands). Crystallite sizes were estimated using the Debye–Scherer equation implemented withing the software [40].

#### 2.2.3. Fourier Transform Infrared Spectroscopy

Fourier Transform Infrared Spectroscopy (FTIR) was performed to analyze Fe and/or Gd powders using Nicolet iS50R (Thermo Fisher, Walthman, MA, USA) equipment. Measurements were conducted at room temperature in attenuated total reflection (ATR) mode. Each sample was scanned 32 times over a wavenumber range of 4000–400 cm^−1^, with a resolution of 4 cm^−1^ [40].

#### 2.2.4. Energy Dispersive X-Ray Spectroscopy

Energy dispersive X-Ray spectroscopy (EDX) was employed to determine the elemental composition of Fe and/or Gd oxide powders. The electron beam was accelerated at 30 kV with a probe current ranging from 1 pA to 300 μA. Quantitative measurements were performed on 20 mg of powder at 5000× magnification, using an electron beam accelerated at 20 kV.

### 2.3. In Vitro Characterization of Cellular Response in 2D Cell Modes in Absence of Irradiation

#### 2.3.1. Cell Culture and Treatment with Nanoparticles

B16 murine melanoma cells and L929 murine fibroblast cells (CLS, Heidelberg, Germany) were cultured in Dulbecco’s Modified Eagle Medium (DMEM) supplemented with 10% fetal bovine serum (FBS) and 1% Penicillin–Streptomycin, under standard incubation conditions (37 °C, 5% CO_2_, 90% humidity).

B16, respectively L929, cells were seeded in 96-well plates at a concentration of 5000 cells/well and incubated for 24 h to allow cell attachment. Subsequently, the culture medium was replaced with nanoparticle-containing medium at various concentrations (0–500 μg/mL), prepared via serial dilution and ultrasonication to ensure homogenous dispersion. Cells were further incubated in the presence of nanoparticles for 48 h under standard conditions, after which cell viability, morphology, and internalization assays were performed.

#### 2.3.2. MTT Tetrazolium Salt Viability Assay

Cell viability and proliferation were evaluated using the tetrazolium salt viability assay MTT (Serva Electrophoresis GmbH, Heidelberg Germany). Following the incubation period, the nanoparticle-containing culture medium was removed and replaced with MTT solution prepared in complete culture medium, followed by incubation for 2 h under standard conditions. This MTT working solution consisted of 10 (*v*/*v*) MTT stock solution (5 mg/mL in PBS) diluted in complete DMEM.

This assay is based on the metabolic reduction of MTT by viable cells to insoluble formazan crystals, with the extent of conversion being proportional to cell viability. After incubation, the MTT-containing medium was removed and formazan crystals were solubilized using dimethyl sulfoxide (DMSO). The absorbance was measured spectrophotometrically at 570 nm.

Blank samples containing nanoparticles at the tested concentrations, but no cells, were prepared, and their absorbance values were subtracted from those of the corresponding cell-containing samples. All experiments were performed three times and in triplicate. Cell viability was calculated by normalizing the absorbance of each sample to that of the untreated control, which is considered 100% viable. Data are presented as the mean ± STDEV (standard deviation), and the statistical evaluation was performed using Student’s *t*-test, with significance levels set at *p* < 0.05 (*), *p* ≤ 0.01 (**), and *p* ≤ 0.001 (***), respectively.

#### 2.3.3. Morphology and Internalization

The morphology of cells following treatment and the internalization of Fe and/or Gd oxide nanoparticles was investigated using scanning electron microscopy (SEM). For this purpose, L929 and B16 cells were cultured on 10 mm glass coverslips and subjected to nanoparticle treatment as described in Section 2.3.1. After the incubation period, non-internalized nanoparticles were removed, and the cells were gently washed several times with phosphate-buffered saline (PBS), followed by fixation with a 1% glutaraldehyde solution in PBS for 1 h at room temperature. The cells were then washed with PBS and dehydrated using ethanol solutions of increasing concentrations (70–100%), followed by ethanol:hexamethyldisilazane solutions at different ratios (50:50, 25:75, and 0:100) for several minutes at room temperature. Subsequently, the samples were coated with a thin layer of gold and analyzed using an SEM microscope. Secondary electron (SE) mode was used to investigate cell morphology and monolayer topography, while backscattered electron (BSE) mode was employed to obtain compositional information.

### 2.4. In Vitro Characterization of Cellular Response Under Ionizing Radiation Conditions in 2D Cell Modes

#### 2.4.1. Cell Culture and Treatment with Nanoparticles

To investigate the biological functional effects of radiosensitization mediated by Fe-Gd nanoparticles in melanoma cells, complementary studies on cell viability and survival were conducted and compared with responses in normal fibroblast cells. Experiments were performed using Fe-Gd nanoparticles containing 10%Gd^3+^, 15%Gd^3+^, and 25% Gd^3+^, and the results were compared with those obtained for Fe_3_O_4_@PEG 6K and Gd_2_O_3_@PEG 6K nanoparticles.

B16 murine melanoma cells and L929 murine fibroblast cells were seeded in 96-well plates at a concentration of 5000 cells/well and incubated under standard conditions to allow cell attachment. After 4 h, the culture medium was removed and replaced with nanoparticle-containing medium at working concentrations of 0 and 200 μg/mL, previously prepared via ultrasonication to ensure a homogenous dispersion. Cells were then incubated in the presence of nanoparticles for 16 h under standard conditions.

Following incubation, the nanoparticle-containing medium was removed, and the cells were thoroughly washed three times with phosphate buffer saline (PBS) to eliminate non-internalized nanoparticles, ensuring that only intracellular nanoparticles remained. The incubation period was selected to remain below the doubling time of the cell lines, thereby preventing dilution of the intracellular nanoparticle content.

Subsequently, fresh nanoparticle-free culture medium was added, and irradiation was performed using an X-Ray generator (X-Strahl XRC, Surrey, UK), operated at 150 kV, with a dose rate of 2.394 mGy/s. Cells were irradiated with doses of 0 or 4 Gy. After irradiation, the cells were incubated for an additional 48 h under standard conditions, after which cell viability and survival were assessed.

#### 2.4.2. MTT Tetrazolium Salt Viability Assay

The assay was performed on cells irradiated in the presence of nanoparticles in order to evaluate the overall metabolic activity and to have an estimate of cell viability. Following the incubation period, the culture medium was removed and replaced with MTT solution prepared as described in Section 2.3.2 and incubated for 2 h under standard conditions. Afterwards, the formazan crystals were solubilized with DMSO and the absorbance was measured at 570 nm. Data processing and statistical analysis was performed according to Section 2.3.2.

#### 2.4.3. ATP Quantification Assay

Intracellular adenosine triphosphate (ATP) levels were quantified to assess cellular metabolic activity following irradiation in the presence of nanoparticles. ATP serves as the primary energy carrier for intracellular processes and reflects mitochondrial and glycolytic activity.

ATP levels were measured using a bioluminescent assay based on the luciferase–luciferin reaction, in which light emission is generated in the presence of ATP and oxygen. When ATP is the limiting reagent, the emitted luminescence is directly proportional to the ATP concentration, with higher luminescence signals indicating increasing ATP levels.

To quantify the ATP production in tumor and normal cells, samples were incubated with a reaction substrate (CellTiter-Glo, Promega, Madison, WI, USA) for 10 min at room temperature. Afterwards, emitted luminescence was measured with a luminometer. All experiments were performed three times and in triplicate. Cell viability was calculated by normalizing the absorbance of each sample to that of the untreated control, which is considered 100% viable. Data are presented as the mean ± STDEV (standard deviation), and the statistical evaluation was performed using Student’s *t*-test, with significance levels set at *p* < 0.05 (*), *p* ≤ 0.01 (**), and *p* ≤ 0.001 (***), respectively.

#### 2.4.4. MitoTracker Test

The mitochondrial content and integrity were evaluated using the MitoTracker Green FM probe (Cell Signaling Technology, Danvers, MA, USA). This assay is based on a fluorescent dye that accumulates in the mitochondria of viable cells, independently of the mitochondrial membrane potential. Fluorescence intensity, measured at an excitation wavelength of 490 nm and an emission wavelength of 516 nm, reflects the total mitochondrial mass and is directly proportional to the mitochondrial content.

A MitoTracker Green FM working solution with final concentration of 100 nM was prepared by diluting 0.6 μL of the stock solution in 6 mL of DMEM. The cell culture medium was replaced with the MitoTracker-containing medium, and the samples were incubated for 45 min under standard conditions. Following incubation, the fluorescence intensity was measured to assess the mitochondrial content.

All experiments were performed three times and in triplicate. Data are presented as the mean ± STDEV (standard deviation). Cell viability and mitochondrial content were normalized to untreated control samples, which were considered 100%. Statistical analysis was performed using Student’s *t*-test, with significance thresholds set at *p* < 0.05 (*), *p* ≤ 0.01 (**), and *p* ≤ 0.001 (***), respectively.

#### 2.4.5. Clonogenic Survival Assay

The clonogenic assay was employed to assess the capacity of a single cell to survive treatment and retain their capacity to proliferate and form colonies. It is considered a gold standard for evaluating cellular radiosensitivity. This assay evaluates long-term cell survival following exposure to nanoparticles and ionizing radiation.

Immediately following irradiation, melanoma and fibroblast cells were detached, counted, and seeded at low concentrations (500 and 1000 cells/well) in 6-well plates. Cells were then incubated for 10 days under standard conditions to allow colony formation. Afterwards, the culture medium was removed, the cells were washed with PBS and colonies were fixed and stained with 0.5% crystal violet in 25% methanol for 30 min. The excess dye was removed, and the colonies were thoroughly washed with water.

Colonies containing more than 50 cells were counted, and the plating efficiency and survival fraction were calculated according to standard protocols [32,33]. All experiments were performed three times and in triplicate. Data are presented as the mean ± STDEV (standard deviation). Statistical analysis was performed using Student’s *t*-test, with significance thresholds set at *p* < 0.05 (*), *p* ≤ 0.01 (**), and *p* ≤ 0.001 (***), respectively.

### 2.5. In Vitro Characterization of Cellular Response in 3D Cell Modes in Absence of Irradiation

#### 2.5.1. Three-Dimensional Multi-Cellular Spheroid Models

Monocellular suspensions were prepared via the enzymatic detachment of B16 murine melanoma and L929 murine fibroblast cells. Three-dimensional multi-cellular models were generated using two approaches, both based on the liquid overlay technique in non-adherent Corning spheroid plates [32].

In the first approach, L929 fibroblast spheroids were generated by seeding cells at densities of 2500 and 5000 cells/well and incubating for 3 days to allow spheroid formation. Subsequently, B16 melanoma cells were added on top of the preformed fibroblast spheroids, at densities of 2500 and 5000 cells/well, followed by an additional 3-day incubation under standard conditions.

The second approach involved the preparation of mixed cell suspensions of B16 and L929 at different ratios, which were directly seeded into non-adherent spheroid plates. All cell suspensions were cultured in complete medium supplemented with murine type I collagen (0.0125–0.05%) (Sigma Aldrich-Merck, Darmstadt, Germany) to promote spheroid stability and extracellular matrix formation.

Spheroids were maintained under standard culture conditions for up to 14 days. The morphology of the resulting spheroids was monitored via optical microscopy every 3–4 days. Images were acquired and quantitatively analyzed using ImageJ software to extract morphological parameters including the mean diameter, perimeter, surface, circularity, aspect ratio, roundness, and solidity. The temporal evolution of spheroid morphology was evaluated to select the optimal 3D model, and the detailed morphological characterization is provided in Supplementary Materials Section S4.

#### 2.5.2. Immunocytochemical Characterization of the 3D Multi-Cellular Models

A subset of the generated spheroids was collected at predefined time points for immunocytochemical characterization. Prior to fixation, spheroids were incubated for 2 h with 30 μM bromodeoxyuridine (BrdU) and 300 μM Pimonidazole (Pimo). Afterwards, spheroids were fixed via overnight incubation at 4 °C in 4% Roti-Histofix (Carl Roth, Karlsruhe, Germany).

Sample dehydration, paraffin embedding, and histological processing were performed according to standard protocols, as detailed in Supplementary Materials Section S4.3. Immunostaining was carried out to identify hypoxic regions using Hypoxyprobe-1 Omni kit PAb 2627(AP) rabbit antisera (Hypoxyprobe, Burlington, MA, USA), proliferative cells using an anti-BrdU rat monoclonal antibody (ab6326 Abcam, Cambridge, UK), melanoma-specific markers using a rabbit monoclonal anti-MelanA antibody (ab51061, Abcam, Cambridge, UK), and malignancy-associated regions using a rabbit monoclonal anti-S100 beta antibody (ab52642, Abcam, Cambridge, UK).

### 2.6. In Vitro Characterization of Cellular Response Under Ionizing Radiation Conditions in 3D Cell Modes

#### Three-Dimensional Multi-Cellular Spheroid Models and Nanoparticle Treatment

Three-dimensional multi-cellular melanoma models were generated using the one-step approach described in Section 2.5.1. Following spheroid formation, the culture medium was replaced with nanoparticle-containing medium, according to the procedure outlined in Section 2.4.1. After 48 h of incubation and the subsequent removal of non-internalized nanoparticles, spheroids were subjected to ionizing radiation treatment.

Spheroid size and morphology were monitored for a period of 7 days following irradiation. Cell viability and metabolic activity were measured using the MTS tetrazolium salt assay and ATP quantification assay, in accordance with the protocols described in Section 2.3.2 and Section 2.4.3. The MTS assay was done using the CellTiter 96 Aqueous Non-Radioactive Cell Proliferation Assay (Promega, Madison, WI, USA) according to the producer’s specifications.

### 2.7. In Vivo Evaluation of Systemic Tolerance and Tissue Response

The in vivo evaluation of systemic tolerance and tissue response studies were conducted in BALB/c mice in compliance with EU Directive 2010/63/EU on the protection of animals used for scientific purposes. All experimental procedures were approved by the Institutional Animal Ethics Committee (No. 113-CECP/8.09.2023). The experiment included five groups, each comprising of five animals, which received an intraperitoneal injection of either physiological saline (control group) or a 25% Gd-doped iron oxide nanoparticle suspension in physiological saline, at a test concentration of 1 mg/mL. Animals were euthanized at predefined time points following administration (24 h, 48 h, 72 h, and 7 days), and the major organs (spleen, liver, kidneys, lungs, myocardium, intestine, and brain) were collected and processed for histological examination, as described in Supplementary Materials, Section S5.

## 3. Results and Discussion

### 3.1. Nanoparticle Synthesis and Characterization

Building upon our previously developed iron oxide nanoparticles [22], which were specifically designed to achieve high internalization and intracellular retention in tumor cells, we developed Gd-doped nanoparticles to be used in the radiosensitization of melanoma cells. The composite nanoparticles were synthesized using a room-temperature co-precipitation method [22], in which varying amounts of the Fe(III) precursor were substituted with Gd (III) precursor. This strategy enabled the incorporation of a high-atomic-number element into the crystalline lattice, thereby enhancing the previously shown radiosensitization efficiency of iron oxide nanoparticles [22,33].

**Figure 1:**
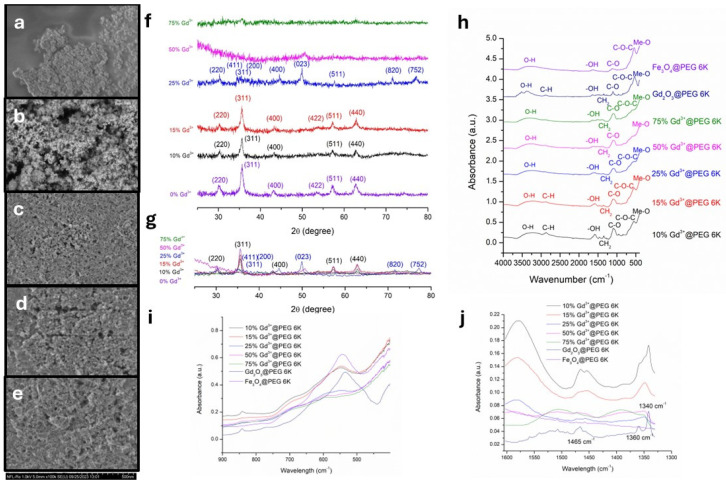
Physicochemical characterization of the nanoparticles. Scanning electron microscopy (SEM) images of (**a**) iron oxide nanoparticles (Fe_3_O_4_@PEG 6K, 0% Gd^3+^), (**b**) gadolinium oxide nanoparticles (Gd_2_O_3_@PEG 6K), and Fe-Gd oxide nanoparticles doped with (**c**) 10% Gd^3+^, (**d**) 15% Gd^3+^, and (**e**) 25% Gd^3+^, respectively. X-Ray diffraction (XRD) patterns of the Fe and/or Gd oxide nanoparticles (**f**,**g**). Fourier-transform infrared (FTIR) spectra of the nanoparticles recorded in the ranges 500–4000 cm^−1^ (**h**), 400–900 cm^−1^ (**i**), and 1300–1600 cm^−1^ (**j**).

The morphology of the synthesized nanoparticles was investigated with SEM (Figure 1a–e and Supplementary Materials, Figures S1–S5). Iron oxide nanoparticles exhibited a predominantly spherical morphology, with an estimated mean diameter of 12.82 ± 2.73 nm (Figure 1a). It should be noted that a certain degree of aggregation was observed, which is typical for Fe oxide nanoparticles, whereas size values were determined from distinguishable individual particles or particle domains.

In contrast, nanoparticles containing 10% Gd^3+^ showcased a predominantly acicular (rod-like) morphology, although isolated populations of spherical nanoparticles were also observed (Figure 1c), likely corresponding to regions with lower Gd incorporation where iron oxide remains the dominant phase. For the 10% Gd sample, the mean particle dimensions were estimated at 47.89 ± 19.09 nm in length and 13.98 ± 2.5 nm in width.

Nanoparticles with 15% Gd^3+^ exhibited an ovoid morphology with an estimated mean size of 18.23 ± 3.95 nm (Figure 1d). Further increasing the Gd^3+^ content to 25% resulted in nanoparticles with an irregular platelet-like morphology and an increased tendency toward aggregation (Figure 1e), similar to that observed for Gd oxide nanoparticles (Figure 1b). Due to the small size, irregular morphology, and aggregation tendency, accurate dimensional analysis of the 25% Gd nanoparticles was limited.

Overall, a morphology transition was observed with an increasing Gd concentration. The incorporation of Gd appears to favor the formation of anisotropic, rod-shaped structures, which may be related to the difference in ionic radii between Gd^3+^ (0.935 Å) and Fe^3+^ (0.645 Å) [41,42].

The XRD diffraction pattern obtained for iron oxide nanoparticles confirmed the presence of diffraction interferences characteristic for the magnetite mineralogic phase in cubic system (Figure 1f,g). The diffraction maxima observed at 2θ = 30.21, 35.58, 43.17, 53.49, 57.25, and 62.75° correspond to the (220), (311), (400), (422), (511), and (440) crystallographic planes, respectively, in accordance with the ICDD card no. 00-007-0322 included in PDF5+ 2025 database.

Upon the incorporation of Gd^3+^ into the nanoparticle composition, systematic changes in the diffraction patterns were observed. For samples containing 10% and 15% Gd^3+^, the characteristic magnetite peaks exhibited a shift toward higher 2θ values (Figure 1f,g and Supplementary Materials Table S1). This shift indicates the lattice distortion induced by Gd^3+^ incorporation. Additionally, a significant increase in crystallite size was observed with an increasing Gd^3+^ content (Supplementary Materials Table S1), suggesting the successful substitution of Fe^3+^/Fe^2+^ ions with Gd^3+^ in a manner proportional to the dopant concentration.

In the case of nanoparticles containing 25% Gd^3+^, the diffraction peaks associated with cubic magnetite were further shifted toward higher values of 2θ and exhibited pronounced peak splitting (Figure 1f,g and Supplementary Materials Table S1), indicating increased structural complexity. Specifically, the (220) plane displayed two maxima at 2θ = 30.24 and 30.32°. The (311) plane exhibited extensive splitting, with peaks at 2θ = 34.34, 34.35, 35.01, 35.57, and 35.66°, which can be attributed not only to the (311) plane, but also to the (411) plane, which is characteristic of cubic Gd_2_O_3_ and the (200) plane associated with gadolinium ferrites [43]. Additional diffraction peaks corresponding to gadolinium ferrite phases were identified at 2θ = 38.01, 38.19, 39.48, and 39.73°. The crystalline plane (400) characteristic for cubic magnetite appeared as a ramified peak with two maxima at 2θ = 43.32 and 43.43°. The peak at 2θ = 42.70° was assigned to the (413) crystalline plane characteristic of Gd_2_O_3_ in the cubic system, and 2θ = 49.95° was assigned to (023) characteristic of gadolinium ferrites [43]. Similarly, the crystalline plane (422) characteristic for cubic magnetite corresponds to a ramified peak with two maxima at 2θ = 57.20 and 57.35°. The crystalline plane (511) characteristic of cubic magnetite corresponds to a ramified peak with two maxima at 2θ = 62.76 and 62.93°. Other diffraction interferences observed at 2θ = 71.52° and 2θ = 77.21° were attributed to (820) and (752) crystalline planes of cubic Gd_2_O_3_ and to the (214) plane of GdO_2_ with tetragonal symmetry, in agreement with the ICDD cards no. 01-080-6925 and 04-003-6351. The increase in crystallite size observed for the 25% Gd^3+^ sample further supports the formation of mixed iron-gadolinium oxide phases (Supplementary Materials Table S1).

For the samples containing 50% Gd^3+^ and 75% Gd^3+^, the XRD patterns revealed a very low degree of crystallinity, preventing the reliable identification of any distinct diffraction peaks (Figure 1f). This behavior suggests the formation of highly disordered or amorphous structures at high Gd concentrations, likely associated with Gd-rich hydroxide phases. The XRD pattern obtained for Gd_2_O_3_ (Supplementary Materials Figure S6) showed diffraction interferences characteristic of both cubic Gd_2_O_3_ and tetragonal GdO_2_, in agreement with ICDD cards no. 01-080-6925 and 04-003-6351. The peaks identified at 2θ = 28.57, 32.05, 40.6, 42.21, 60.23, 62.92, 73.32, 76.165, 77.97, and 78.92° correspond to the crystalline planes of (222), (111), (422), (112), (543), (633), (822), (622), (311), and (048) characteristic of the Gd_2_O_3_ in the cubic system. The peaks identified at 2θ = 26.23, 26.75, 35.23, 59.16, and 65.14° correspond to the crystalline planes of (101), (002), (102), (212), and (114) characteristic of the GdO_2_ crystalline phase in a tetragonal system. Several peaks including those at 2θ = 45.95, 48.02, 52.6, 53.66, and 66.98° can be attributed to both cubic Gd_2_O_3_ and tetragonal GdO_2_ phases.

The FTIR spectrums of Fe-Gd oxide nanoparticles are presented in Figure 1h and were vertically offset to enhance graphical clarity. The complex absorption band observed in the spectral region below 700 cm^−1^ is characteristic of metal oxide stretching vibrations [44] with the 400–600 cm^−1^ region being specifically attributed to metal-oxygen (Me-O) bonds [43,44]. In this study, distinct spectral modifications were observed in the 490–590 cm^−1^ region, which can be ascribed to the substitution of Fe^3+^ with Gd^3+^ within the nanoparticle lattice (Figure 1i).

For Gd oxide nanoparticles, a characteristic absorption maximum was identified at 533 cm^−1^, while Fe oxide nanoparticles exhibited a corresponding peak at 544 cm^−1^. Upon substitution with 10%Gd^3+^, this peak shifted towards the right area of the spectrum, reaching 548 cm^−1^, and further shifted to 549 cm^−1^ for the 15% Gd^3+^ substitution. With an increasing Gd content, the absorption maximum progressively shifted toward 550, 598, and 599 cm^−1^, accompanied by a significant band broadening (Figure 1i). Such peak shifts and broadening are indicative of lattice distortions and defect formation in metal oxides induced by cation substitution [43,44].

According to Azeem et al. [43], the spectral region between 1300 and 1600 cm^−1^ represents a characteristic fingerprint of gadolinium oxide. In the FTIR spectrums of iron oxide nanoparticles, this region exhibited minimal modulations, whereas distinct absorption bands were observed for Gd oxide nanoparticles and Fe-Gd oxide composites (Figure 1j). For Gd oxide nanoparticles, characteristic peaks were identified at 1340 cm^−1^, 1360 cm^−1^, and 1465 cm^−1^. In the case of Fe-Gd oxide nanoparticles, the band at 1340 cm^−1^ broadened with an increasing Gd^3+^ content, progressively overlapping the peak at 1360 cm^−1^, while its intensity decreased. Additionally, the absorption peak at 1465 cm^−1^ shifted toward the right area of the spectrum and exhibited increased broadening with higher Gd concentrations. For Fe-Gd nanoparticles containing the highest Gd^3+^ fractions (25%, 50%, and 75%), a marked decrease in the intensity of this band was observed (Figure 1j). EDX spectroscopy confirmed the presence of Fe, O, and Gd elements in the obtained samples (Supplementary Materials Figure S7).

Successful conjugation of the nanoparticles with PEG 6K was confirmed by the presence of characteristic functional group vibrations (Figure 1h). Deformation vibrations of methylene (-CH_2_-) groups were observed at approximately 1340 cm^−1^ and 1460 cm^−1^, while O-H bending vibrations appeared near 1580 cm^−1^. Stretching vibrations of hydroxyl groups were identified in the spectral region between 3200 and 3500 cm^−1^. Furthermore, the C-O stretching vibrations characteristic of PEG were identified at wavelengths between 1050 and 1100 cm^−1^, and C-H stretching vibrations were observed in selected samples at wavelengths between 2800 and 3000 cm^−1^ [45,46].

### 3.2. In Vitro Characterization of Cellular Response in 2D Cell Modes in Absence of Irradiation

The cell viability assay determinations in the absence of ionizing radiation demonstrated the biocompatibility of the synthesized Fe-Gd oxide nanoparticles in both investigated cell models, namely B16 murine melanoma cells and L929 normal murine fibroblasts. Importantly, under non-irradiated conditions, cell viability did not decrease below the generally accepted biocompatibility threshold of 70% relative to the negative control after 48 h exposure. These results indicate a favorable cytocompatibility profile of Fe-Gd oxide nanoparticles across the tested concentration range.

**Figure 2:**
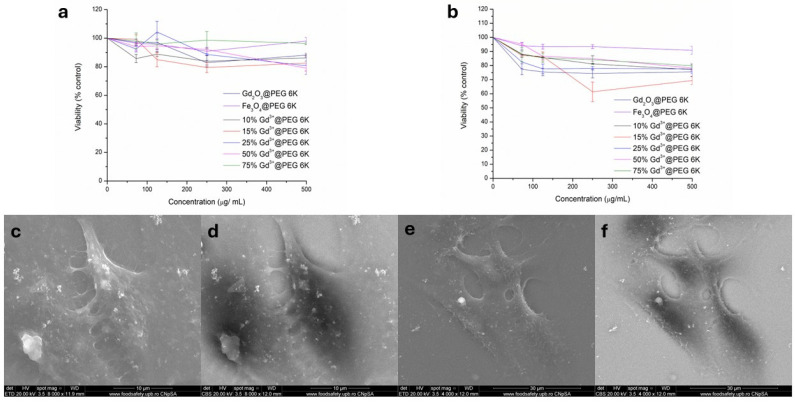
In vitro characterization of cellular response in 2D cell modes in the absence of irradiation. Cell viability assessed based on the MTT assay in (**a**) B16 murine melanoma cells and (**b**) L929 murine fibroblast cells following 48 h exposure to Fe and/or Gd oxide nanoparticles. Representative scanning electron microscopy (SEM) images of B16 cells treated with 25% Gd^3+^ @ PEG 6K for 48 h are shown in secondary electrons mode (**c**,**e**) and backscattered electrons mode (**d**,**f**). Images were acquired at magnifications of 8000× (**c**,**d**) and 4000× (**e**,**f**). Data are expressed as the mean ± STDEV (*n* = 3).

In B16 melanoma cells, all Fe and/or Gd oxide nanoparticle formulations exhibited good biocompatibility, with cell viability remaining above 80% for all investigated concentrations (Figure 2a). No statistically significant cytotoxic effects were observed, indicating that both iron oxide and Gd-doped nanoparticles were well tolerated by the tumor cell line in the absence of ionizing radiation.

Similar effects were observed for L929 fibroblast cells (Figure 2b). In this case, cell viability values were slightly lower than those recorded for B16 cells, with an approximate decrease of 10%, which was statistically non-significant (NS). This effect was more pronounced at higher nanoparticle concentrations (above 250 μg/mL), particularly for the 25% Gd^3+^-doped formulation. The observed reduction in L929 cell viability correlated with increasing concentrations of Gd-containing nanoparticles, consistent with the known dose-dependent concentration of Gd^3+^ ions [41]. Nevertheless, all viability values remained within the accepted biocompatibility range for all nanoparticle concentrations evaluated in this study.

### 3.3. In Vitro Characterization of Cellular Response Under Ionizing Radiation Conditions in 2D Cell Modes

**Figure 3:**
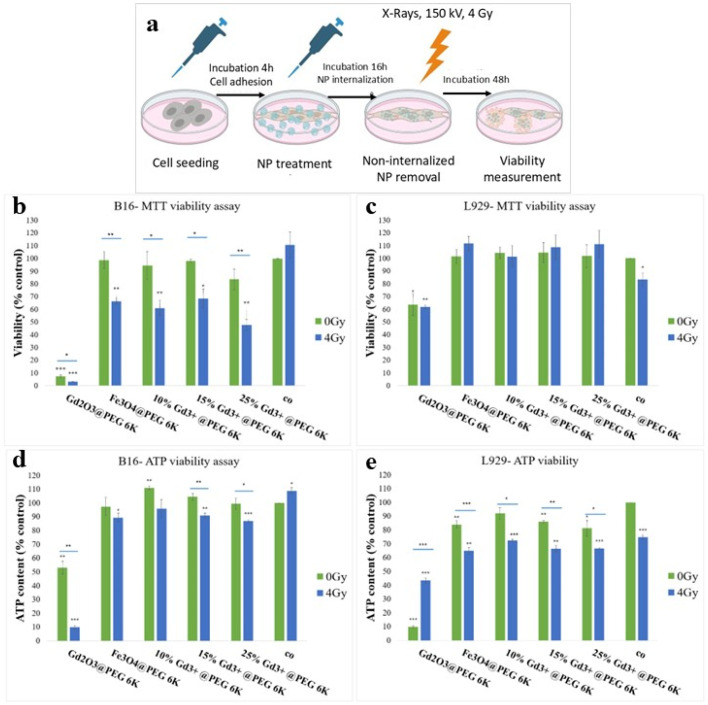
In vitro characterization of cellular response under ionizing radiation conditions in 2D cell modes. Schematic representation of the treatment protocol (**a**). Cell viability assessed based on the MTT assay (**b**,**c**) and by ATP assay (**d**,**e**) in (**b**,**d**) B16 murine melanoma cells and (**c**,**e**) L929 murine fibroblast cells following 48 h exposure to Fe and/or Gd oxide nanoparticles. Data are expressed as the mean ± STDEV (*n* = 3), where *p* < 0.05 (*), *p* ≤ 0.01 (**) and *p* ≤ 0.001 (***).

Based on the physicochemical characteristics, particularly XRD evidence of structural changes at higher Gd contents, as well as the biological response observed in the absence of ionizing radiation in L929 and B16 cell lines, experiments in the presence of ionizing radiation were performed on selected iron oxide nanoparticles doped with 10%, 15%, and 25% Gd^3+^, in comparison with non-doped Fe and Gd oxide nanoparticles. Cell viability was evaluated 48 h after the X-Ray irradiation (150 kV, 4 Gy) in cells that had been previously incubated with nanoparticles at a concentration of 200 μg/mL for 16 h (Figure 3a). This incubation interval was selected as it is shorter than the doubling time of both B16 and L929 cell lines, thereby minimizing the dilution of the intracellular nanoparticle content due to cell division. Following incubation, non-internalized nanoparticles were removed via washing, prior to irradiation (Figure 3a). The nanoparticle concentration was chosen based on its demonstrated non-cytotoxic behavior, efficient cellular internalization, and previously reported radiosensitization efficiency in similar experimental models [30,31,32,33].

Cell viability was assessed using the tetrazolium salt (MTT) assay, which reflects the overall metabolic activity. The reduction of the tetrazolium salts is primarily mediated by mitochondrial electron transport during the glycolysis process, but it is also influenced by enzymatic and redox processes occurring in the endoplasmic reticulum, cytoplasm, plasma membrane, microsomes, and nucleus [47,48]. Therefore, cell viability values expressed relative to the negative control are an integrated measure of how the applied treatments affect total cellular metabolism. In melanoma B16 cells, the results confirmed the biocompatibility of Fe and Fe-Gd oxide nanoparticles under non-irradiated conditions, as MTT-based viability measurements indicated no significant impairment of cellular metabolism (Figure 3b). Upon exposure to ionizing radiation, a decrease in MTT-derived metabolic activity was observed compared both to the negative control and to the corresponding non-irradiated samples (Figure 3b). Specifically, metabolic activity was reduced with the following: 25.72 ± 4.19% for 4 Gy Fe_3_O_4_@PEG 6K compared to 0 Gy Fe_3_O_4_@PEG 6K (*p* = 0.01); 27.39 ± 4.87% for 4 Gy 10% Gd^3+^@ PEG 6K versus 0 Gy 10% Gd^3+^@ PEG 6K, (*p* = 0.03); 22.24 ± 6.13% for 4 Gy 15% Gd^3+^@ PEG 6K versus 0 Gy 15% Gd^3+^@ PEG 6K (*p* = 0.03); 30.63 ± 3.68% for 4 Gy 25% Gd^3+^@ PEG 6K versus 0 Gy 25% Gd^3+^@ PEG 6K (*p* = 0.009) (Figure 3b). In contrast, melanoma cells exposed to 200 µg/mL Gd_2_O_3_@ PEG 6K exhibited a pronounced cytotoxic response even in the absence of ionizing radiation (Figure 3b), indicating the intrinsic toxicity of gadolinium oxide [49,50] nanoparticles, rather than a radiosensitization-driven effect. In the case of L929 fibroblast cells, exposure to Fe and/or Gd oxide nanoparticles followed by ionizing radiation treatment slightly altered the cell metabolic activity; however, the response did not show any cytotoxic effect (Figure 3c). On the other hand, the treatment with Gd oxide nanoparticles induced a reduction in cell overall metabolism with about 60% relative to the negative control, indicative of a moderate cytotoxic effect. Notably, subsequent exposure to ionizing radiation did not significantly exacerbate this response (Figure 3c).

Unlike the MTT, the luminescence-based ATP quantification assay selectively evaluates the mitochondrial metabolic activity. This method relies on the enzymatic conversion of D-luciferin to oxyluciferin, catalyzed by luciferase, in the presence of adenosine triphosphate (ATP) [51]. The amount of oxyluciferin produced, which generates the luminescent signal, is directly proportional to the intracellular ATP concentration and therefore serves as a quantitative indicator of mitochondrial metabolism [52,53]. In irradiated melanoma B16 cells (Figure 3d), a statistically significant increase in ATP production was measured compared to the negative control. This effect is likely associated with a radiation-induced adaptive response, rather than enhanced cell survival. A transient increase in metabolic activity (also measured in the MTT-based assay, although not significant) may reflect a hermetic stress-related response in relation to the activation of cellular repair mechanisms [54,55,56].

Nevertheless, the treatment with Fe-Gd nanoparticles did not significantly influence ATP production, compared to the negative control. Notably, for the composite nanoparticles containing the highest Gd^3+^ concentration (10% Gd^3+^ @ PEG 6K), a statistically significant increase in ATP levels was noticed compared to the negative control (with 11 ± 1.12%, *p* = 0.003), suggesting mild metabolic stimulation (Figure 3d). A substantial decrease in ATP production was observed exclusively in the case of melanoma cells exposed to Gd oxide nanoparticles, with ATP levels reduced at 53.22 ± 4.6% (*p* = 0.003 compared to untreated cells). The melanoma cells exposed to the combined treatment (nanoparticles and irradiation) showcased a reduction in ATP production, compared to cells treated with nanoparticles alone. Specifically, ATP levels decreased significantly by 12.48 ± 3.21% (*p* = 0.03) in cells treated with the Fe-Gd nanocomposite containing the highest Gd content (10% Gd^3+^ @ PEG 6K), by 13.67 ± 0.632 (*p* = 0.002) in cells treated with 15% Gd^3+^ @ PEG 6K, and by 43.18 ± 3.41% (*p* < 0.01) in cells treated with Gd oxide nanoparticles, followed by irradiation, relative to the corresponding non-irradiated samples.

In L929 fibroblast cells (Figure 3e), ATP levels did not decrease by more than 20% relative to the negative control following the exposure of the cells to Fe and Fe-Gd oxide nanoparticles combined with ionizing radiation, indicating preserved mitochondrial metabolic activity. In contrast, cells exposed to Gd oxide nanoparticles exhibited a pronounced reduction in ATP content, reaching 10.08 ± 0.67% of the negative control (*p* < 0.001). When Gd oxide nanoparticle exposure was combined with irradiation, ATP levels reached 43.54 ± 1.9 of the negative control (*p* < 0.001). A slight tendency toward decreased ATP production was observed following exposure to 4 Gy X-Rays, in comparison to nanoparticle treatment alone; however, this trend was statistically significant for all investigated samples (*p* < 0.05).

To further validate the statistical relevance of these observations, an extended statistical analysis was performed using one-way ANOVA followed by Tukey’s multiple comparison test and two-way ANOVA. In B16 melanoma cells cultured in 2D conditions, two-way ANOVA revealed significant effects of nanoparticle exposure (*p* < 0.001) and irradiation (*p* < 0.001) on cellular viability, together with a significant interaction between nanoparticle treatment and irradiation (*p* = 0.00295), indicating that the cellular response to irradiation depended on nanoparticle exposure. One-way ANOVA further confirmed significant differences among treatment groups (F = 98.55, *p* < 0.001). Tukey’s multiple comparison test indicated that several nanoparticle formulations combined with irradiation produced significantly lower viability compared with the irradiated control, particularly for the 25% Gd, 15% Gd, and 10% Gd and Fe oxide nanoparticle formulations, supporting their radiosensitizing effect in melanoma cells. In contrast, under non-irradiated conditions, Fe oxide and Fe-Gd oxide nanoparticles maintained viability levels close to the untreated control, whereas Gd oxide nanoparticles induced a marked reduction in viability irrespective of irradiation, indicating the intrinsic cytotoxicity of this formulation. In L929 fibroblast cells cultured in 2D conditions, two-way ANOVA also revealed significant effects of nanoparticle exposure (*p* < 0.001) and irradiation (*p* < 0.001) on cellular viability; however, no significant interaction between these factors was detected (*p* = 0.702), indicating that the cellular response to irradiation did not depend on nanoparticle exposure in normal fibroblasts. One-way ANOVA confirmed significant differences among treatment groups (F = 17.02, *p* < 0.001), with Tukey’s test indicating that these differences were mainly driven by the markedly reduced viability observed for Gd oxide nanoparticles under both irradiated and non-irradiated conditions. In contrast, Fe oxide and Fe-Gd oxide nanoparticle formulations maintained viability levels comparable to the untreated control, supporting their favorable biocompatibility toward normal fibroblasts.

A similar statistical approach was applied to ATP measurements evaluating mitochondrial metabolism. In B16 melanoma cells, two-way ANOVA revealed significant effects of nanoparticle exposure (*p* < 0.001) and irradiation (*p* < 0.001), together with a significant interaction between these factors (*p* < 0.001), indicating that the energetic response of melanoma cells to irradiation depended on nanoparticle treatment. One-way ANOVA further confirmed significant differences among treatment groups (F = 191.74, *p* < 0.001). Tukey’s multiple comparison test showed that irradiation alone significantly reduced ATP levels compared with the non-irradiated control, while several nanoparticle formulations further amplified this energetic impairment under irradiation conditions. In particular, Fe oxide and Fe-Gd oxide nanoparticle treatments combined with irradiation resulted in significantly lower ATP levels compared with the irradiated control. The most pronounced energetic disruption was observed for Gd oxide nanoparticles, which induced a marked reduction in ATP levels both in irradiated and non-irradiated conditions, indicating the strong intrinsic cytotoxicity of this formulation toward melanoma cells. In L929 fibroblast cells, two-way ANOVA also revealed significant effects of nanoparticle exposure (*p* < 0.001) and irradiation (*p* < 0.001) on mitochondrial metabolism, together with a significant interaction between these factors (*p* < 0.001). One-way ANOVA confirmed significant differences among treatment groups (F = 267.54, *p* < 0.001), and Tukey’s test indicated that several nanoparticle formulations combined with irradiation significantly reduced ATP levels compared with the irradiated control. Again, the strongest energetic impairment was observed for Gd oxide nanoparticles, which induced a pronounced reduction in ATP levels under both irradiated and non-irradiated conditions.

The analysis of the cell viability assay using tetrazolium salt-based assay and quantitative ATP assay, indicates that, in the case of fibroblast cells, viability is mostly maintained within biocompatibility limits for samples exposed to Fe and Fe-Gd oxide and/or X-Rays. These cells exhibit a tendency to accelerate their metabolic activity following irradiation, likely as a compensatory response to support the cellular repair mechanisms. In contrast, melanoma tumor cells display a reduction in cellular metabolism following combined treatment (nanoparticles and irradiation). The total cellular metabolism decreased down to 30% in samples subjected to combined treatment compared to those exposed to nanoparticles alone (*p* < 0.05), while ATP production decreased with approximately 14% under the same conditions (*p* < 0.05). These findings indicate a differential metabolic response between normal fibroblasts and melanoma cells, suggesting a radiosensitization-mediated impairment of mitochondrial function in tumor cells. The differential response of tumor versus normal cells to combined treatment (nanoparticles and irradiation) can be explained by the Warburg effect [57,58,59,60]. This phenomenon, characteristic of cancer cells, involves a predominant reliance on anaerobic metabolism, whereby glucose is converted in the cytoplasm to pyruvate and subsequently to lactate (or ethanol) via fermentation pathways. Although energetically inefficient in terms of ATP generation, this metabolic reprogramming supports rapid proliferation and may arise from hypoxic conditions within the tumor microenvironment or from partial or complete mitochondrial dysfunction, with an important role in inhibiting the cellular apoptosis in tumorigenesis.

**Figure 4:**
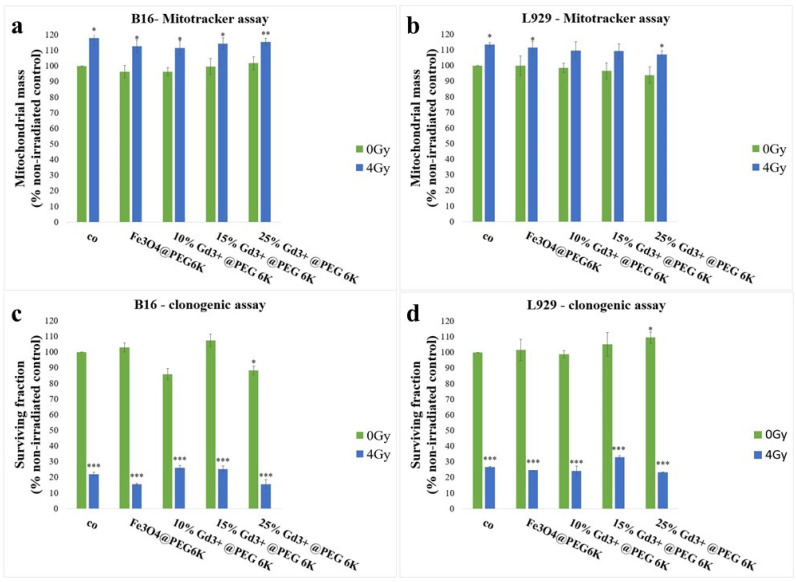
Mitochondrial functionality insight into the cellular response under ionizing radiation conditions. Mitochondrial mass measured with Mitotracker assay in (**a**) B16 murine melanoma cells and (**b**) L929 murine fibroblast cells following 48 h exposure to Fe and/or Gd oxide nanoparticles and irradiation. Surviving fraction of treated cells (**c**) B16 and (**d**) L929 measured using the clonogenic assay. Data are expressed as the mean ± STDEV (*n* = 3), where *p* < 0.05 (*), *p* ≤ 0.01 (**), and *p* ≤0.001 (***).

Further evaluation of mitochondrial alterations associated with the radionsensitizing effects of Fe and/or Gd oxide nanoparticles in melanoma cells were performed using the MitoTracker Green assay, in order to assess changes in total mitochondrial mass following nanoparticle exposure and/or irradiation (Figure 4a,b). A radiation-induced increase in mitochondrial mass was observed in B16 melanoma cells (Figure 4a). In this case, irradiation alone resulted in a 17.8% increase in fluorescence intensity compared with the negative control (*p* = 0.022). Exposure to nanoparticles alone did not significantly affect the mitochondrial mass in melanoma cells; however, the combined treatment (nanoparticles followed by irradiation) significantly increased the fluorescence intensity compared with both the negative control and nanoparticle-only samples for all investigated formulations (*p* < 0.05, Figure 4a). Notably, this effect was not dependent on the Gd content. Thus, B16 cells exposed to Fe_3_O_4_@PEG 6K followed by irradiation exhibited an increase in mitochondrial mass of 12.53% compared with the negative control (*p* = 0.033) and 16.16% compared with nanoparticles alone (*p* = 0.007). In melanoma cells treated with Fe-Gd nanoparticles containing the lowest Gd concentration, the mitochondrial content increased by 11.6% relative to the negative control (*p* = 0.04) and by 15.27% relative to nanoparticles alone (*p* = 0.011). For nanoparticles with the highest Gd concentration, the mitochondrial mass increased by 15.32% compared with the negative control (*p* = 0.007) and by 13.63% compared with nanoparticles alone (*p* = 0.013). Other studies have reported an increase in mitochondrial mass after irradiation in cancer cells, a response commonly associated with the activation of mitochondrial biogenesis [56], a survival-promoting behavior triggered in response to stress [61,62]. In the present study, melanoma cells exposed to Fe and/or Gd oxide nanoparticles followed by ionizing radiation also exhibited an increased mitochondrial mass, an effect mainly attributed to radiation treatment, as no significant difference was observed between irradiation alone and combined nanoparticle-irradiation treatment (Figure 4a). However, despite this increase in mitochondrial mass, ATP production was reduced in cells receiving combined treatment (Figure 3d), indicating impaired mitochondrial energetic function. These findings suggest that the observed increase in mitochondrial mass reflects a stress-adaptive response rather than an improved mitochondrial function, consistent with an imbalance between the mitochondrial biogenesis and energetic behavior.

To further validate the statistical relevance of these observations, an extended statistical analysis was performed using one-way ANOVA followed by Tukey’s multiple comparison test and two-way ANOVA. In B16 melanoma cells cultured in 2D conditions, two-way ANOVA revealed that irradiation had a significant effect on the mitochondrial mass (*p* < 0.001), whereas nanoparticle exposure alone did not produce a significant effect (*p* = 0.113), and no significant interaction between nanoparticle treatment and irradiation was observed (*p* = 0.902). These results indicate that the modulation of mitochondrial mass was primarily driven by irradiation rather than by nanoparticle exposure. One-way ANOVA further confirmed significant differences among treatment groups (F = 15.02, *p* < 0.001). Tukey’s multiple comparison test showed that all irradiated groups exhibited significantly increased mitochondrial mass compared with the corresponding non-irradiated conditions, while no significant differences were observed between nanoparticle formulations within the same irradiation condition.

In irradiated L929 fibroblast cells, fluorescence intensity increased by 13.33% compared with the negative control (*p* < 0.05, Figure 4b), indicating a significant increase in mitochondrial mass as a cellular response to ionizing radiation [63,64]. In fibroblast cells exposed to nanoparticles alone, a slight decrease in mitochondrial mass was observed, proportional to the Gd content. However, this effect was not statistically significant relative to the negative control. In contrast, the application of combined treatment (nanoparticles followed by irradiation) resulted in a significant increase in mitochondrial mass in fibroblast cells compared with both the negative control and nanoparticle-only samples for all investigated formulations (Figure 4b). Overall, these results indicate that normal fibroblast cells responded to radiation and combined nanoparticle–radiation treatment by increasing their mitochondrial mass, without a concomitant impairment of energetic capacity, suggesting an adaptive or compensatory mechanism. In contrast, although B16 melanoma cells also exhibited an increase in total mitochondrial mass following irradiation or dual treatment, this response did not compensate for the treatment-induced damage, leading to significant alterations in overall cellular metabolism and energetic capacity.

To further validate the statistical relevance of these observations, an extended statistical analysis was performed using one-way ANOVA followed by Tukey’s multiple comparison test and two-way ANOVA. In L929 fibroblast cells cultured in 2D conditions, two-way ANOVA revealed that irradiation had a significant overall effect on mitochondrial mass (*p* < 0.001), whereas nanoparticle exposure alone did not produce a significant effect (*p* = 0.142), and no significant interaction between nanoparticle treatment and irradiation was observed (*p* = 0.988). These results indicate that the modulation of mitochondrial mass was primarily driven by irradiation rather than by nanoparticle exposure. One-way ANOVA further confirmed significant differences among treatment groups (F = 7.85, *p* < 0.001). Tukey’s multiple comparison test showed that irradiation increased the mitochondrial mass relative to the untreated control and several non-irradiated conditions. When comparing identical nanoparticle treatments, a significant increase in mitochondrial mass after irradiation was observed for the 15% and 25% Gd-containing nanoparticle formulations, whereas the differences for Fe oxide and 10% Gd nanoparticles were not statistically significant.

The clonogenic test assesses the long-term cell survival and division capacity, representing a more stringent biological endpoint compared to metabolic viability assays. In B16 melanoma cells, the combined nanoparticle–irradiation treatment resulted in an additional reduction in clonogenic survival compared with irradiation alone, demonstrating a radiosensitizing effect for the selected nanoparticle formulations (Figure 4c). Irradiation with 4 Gy X-Rays alone reduced the colony-forming ability of melanoma cells by 78.15% relative to the negative control (*p* < 0.001). Treatment with undoped Fe oxide nanoparticles alone did not significantly affect clonogenic survival, whereas exposure to Gd-doped nanoparticles induced a moderate but statistically significant decrease in survival, independent of the Gd concentration (*p* < 0.05, Figure 4c). The combination of Fe_3_O_4_@PEG 6K nanoparticles and irradiation led to a statistically significant additional reduction in clonogenic survival, by 6.35% compared with irradiated cells (*p* < 0.001) and 87.4% compared with nanoparticle-treated (*p* = 0.006), non-irradiated cells, confirming the radiosensitizing potential of Fe oxide nanoparticles in melanoma cells (Figure 4c). Although Fe-Gd oxide nanoparticles containing 10% and 15% Gd produced a significant decrease in clonogenic survival compared with untreated and nanoparticle-only controls, no statistically significant difference was observed relative to irradiation alone, and therefore, a radiosensitizing effect could not be confirmed for these formulations. In contrast, melanoma cells exposed to nanoparticles containing 25%Gd followed by irradiation exhibited enhanced clonogenic inactivation, with survival reduced by 94.5% compared to untreated control cells, 72.5% compared to nanoparticle-only treated cells, and 6.3% compared to cells irradiated with 4 Gy alone (*p* < 0.001, Figure 4c).

To further validate the statistical relevance of these observations, an extended statistical analysis was performed using one-way ANOVA followed by Tukey’s multiple comparison test and two-way ANOVA. In B16 melanoma cells cultured in 2D conditions, two-way ANOVA revealed significant effects of nanoparticle exposure (*p* < 0.001) and irradiation (*p* < 0.001) on clonogenic survival, together with a significant interaction between nanoparticle treatment and irradiation (*p* < 0.001), indicating that the long-term proliferative response to irradiation depended on nanoparticle exposure. One-way ANOVA further confirmed significant differences among treatment groups (F = 863.67, *p* < 0.001). Tukey’s multiple comparison test showed that all irradiated conditions exhibited markedly reduced clonogenic survival compared with the corresponding non-irradiated groups. Previous studies have shown that nanoparticle-based radiosensitization can significantly reduce the clonogenic survival of melanoma cells, effects frequently associated with mitochondrial dysfunction. For example, cobalt oxide nanoparticles were reported to induce concentration-dependent cytotoxicity in A-375 melanoma cells, an effect linked to the dysregulation of mitochondrial gene expression [65]. In addition, in silico studies have suggested that effective radiosensitization might be achieved by cytoplasm-localized nanoparticles through mechanisms involving mitochondrial DNA damage [66]. Moreover, Chen Y. et al. [67] investigated Gd-doped Ti-based nanoparticle radiosensitizers in a breast cancer model and showed that the reduced clonogenic survival observed after combined nanoparticle–irradiation treatment was associated with reactive oxygen species-mediated apoptotic signaling pathways. In line with these reports, the present study confirmed that Fe and/or Gd- based nanoparticles enhanced the radiation-induced clonogenic inhibition in B16 melanoma cells, with the observed effects being associated with alterations in mitochondrial function.

In the case of L929 normal fibroblast cells, irradiation alone with 4 Gy X-Rays induced a significant reduction in cell survival (Figure 4d), in agreement with previously reported data on this type of cell [68,69]. Exposure to undoped Fe oxide nanoparticles or to Fe-Gd oxide nanoparticles with the lowest Gd content did not induce statistically significant changes in clonogenic survival compared to the negative control. In contrast, nanoparticles containing 15% Gd and 25% Gd produced a modest but significant inhibition of fibroblast clonogenic survival (approximately 5% and 9.4%, respectively; *p* < 0.05). However, the combined treatment (nanoparticles followed by irradiation) did not result in a further significant decrease in survival compared with irradiation alone, indicating the absence of a radiosensitizig effect of Fe- and/or Gd-based nanoparticles in L929 cells under investigated conditions (Figure 4d). To further validate the statistical relevance of these observations, an extended statistical analysis was performed using one-way ANOVA followed by Tukey’s multiple comparison test and two-way ANOVA. In L929 fibroblast cells cultured in 2D conditions, two-way ANOVA revealed significant effects of nanoparticle exposure (*p* = 0.0158) and irradiation (*p* < 0.001) on clonogenic survival, together with a significant interaction between nanoparticle treatment and irradiation (*p* = 0.0262), indicating that the long-term proliferative response to irradiation depended on nanoparticle exposure. One-way ANOVA further confirmed significant differences among treatment groups (F = 366.99, *p* < 0.001).

Although the difference in clonogenic response between normal fibroblasts and melanoma cells was not highly pronounced, the observed effects remain biologically relevant and can be attributed, at least in part, to the use of a relatively high single radiation dose. The selection of the 4 Gy dose was based on previous studies demonstrating a radiosensitizing effect of the Fe_3_O_4_@PEG 6K nanoparticle formulation in cervical adenocarcinoma cells at this dose [32,33]. Importantly, the present clonogenic data are supported by metabolic assays and mitochondrial mass measurements, which indicate distinct functional mitochondrial responses of normal versus tumor cells to the combined treatment. While fibroblast cells retain a compensatory metabolic mechanism despite reduced clonogenic survival following irradiation, melanoma cells exhibit the concomitant impairment of energetic metabolism and long-term proliferative capacity following nanoparticle-mediated radiosensitization.

### 3.4. In Vitro Characterization of Cellular Response Under Ionizing Radiation Conditions in 3D Cell Modes

The next step involved investigating these effects in more complex cellular models with increased in vivo relevance. For this purpose, 3D melanoma cell models were obtained. A key advantage of 3D cell cultures over conventional 2D models is their ability to elicit physiologically relevant cellular responses in experiments evaluating the nanoparticle–cell interactions [70,71]. In particular, 3D cell models provide more realistic information regarding nanoparticle tissue penetrability and cellular internalization under simulated in vivo-like conditions [72,73]. Cells benefit from the 3D architecture through an organization of the extracellular matrix (ECM) that more closely resembles native tissue. Furthermore, this spatial organization promotes the secretion of ECM-specific proteins, thereby enhancing multidirectional intercellular interactions. Such conditions stimulate the heterogenicity of the model, not only in multi-cellular cultures, but also in mono-cellular cultures. Due to their malignant and highly invasive phenotype, B16 melanoma cells do not spontaneously form a compact 3D structure with a well-regulated morphology (Supplementary Materials Figure S9), which complicates their manipulation and limits the reliable assessment of anticancer treatment strategies. Consequently, the proposed 3D model employed L929 fibroblast cells, not only for their ability to generate ECM and provide structural support to the melanoma cells, but also because of their natural presence within the tumor microenvironment. Several approaches and compositions were evaluated (Supplementary Materials Section S4); however, the optimal model in terms of a homogenous spheroid shape and compaction was obtained via the simultaneous seeding of unequal cell ratios (either 2500/5000 L929 cells and 5000/2500 B16 cells) in the presence of 0.05% type I collagen.

Moreover, the cultivation of cells in a spheroid architecture facilitates the establishment of physiological gradients of oxygen, nutrients, and signaling molecules, which are characteristic of living tissues [32,73]. Particularly, in tumor-derived 3D cell cultures, this feature promotes the development of a biomimetic microenvironment, driven by the spontaneous formation of oxygen and nutrient gradients within the spheroids. As a result, distinct phenotypic behaviors representative of in vivo conditions can emerge, including the formation of a peripheral proliferative zone, which was confirmed in our spheroids via BrdU labeling (Supplementary Materials Figure S10). However, the presence of hypoxic regions was not evident, most likely due to the relatively medium-loose morphological organization of the spheroids (Supplementary Materials Figure S11). Nevertheless, the 3D models expressed melanoma-specific biomarkers associated with malignancy and invasiveness, including MelanA and S100 proteins (Supplementary Materials Figure S12).

The selected spheroid models were incubated with nanoparticles for 48 h prior to the removal of non-internalized material and subsequent irradiation. This incubation timeframe is consistent with our previous investigations regarding optimal nanoparticle penetration into 3D spheroid structures [32]. In such models, nanoparticle transport occurs predominantly through passive diffusion, a static mechanism that closely resembles in vivo conditions [32,70]. This behavior is a consequence of tumor-like characteristics, including a dense tissue architecture, abnormal vasculature, and high interstitial fluid pressure, which collectively limit convective transport and render diffusion the dominant mechanism governing the penetration of large molecules and nanoparticles [74,75,76].

**Figure 5:**
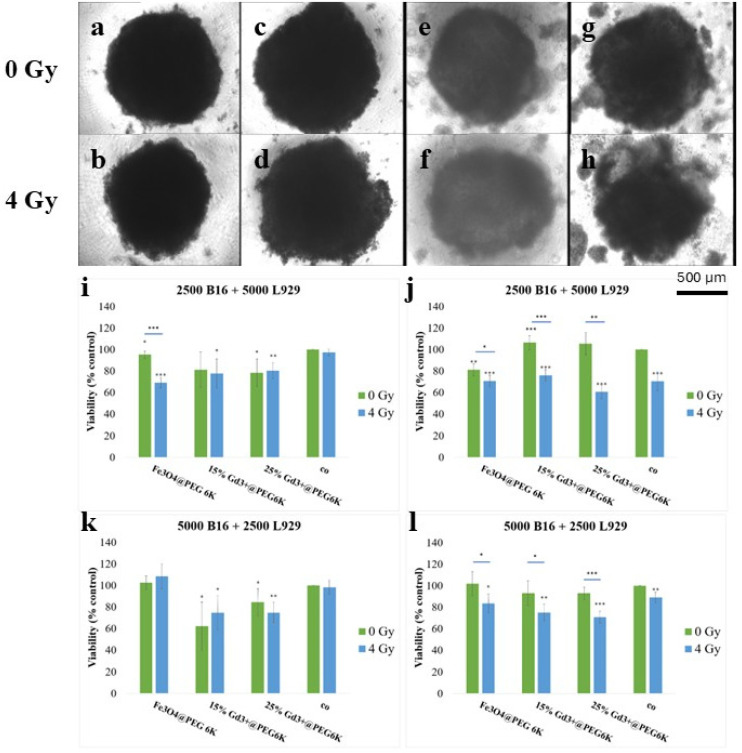
In vitro characterization of cellular response under ionizing radiation conditions in 3D cell modes. Spheroids morphology (5000 B16 + 2500 L929) after 7 days of treatment: (**a**,**b**) control, (**c**,**d**) Fe_3_O_4_ @PEG 6K, (**e**,**f**) 15% Gd^3+^ @PEG 6K, (**g**,**h**) 25% Gd^3+^ @PEG 6K. Cellular viability assessed using the MTS assay (**i**,**k**) and ATP assay (**j**,**l**). Data are expressed as the mean ± STDEV (*n* = 3), where *p* < 0.05 (*), *p* ≤ 0.01 (**), and *p* ≤ 0.001 (***).

Exposure to ionizing radiation did not significantly alter the overall dimension of the 3D melanoma models (5000 B16 + 2500 L929 model) compared with the non-irradiated control (Figure 5a–h). Similarly, the application of the dual treatment (nanoparticles followed by irradiation) did not induce significant changes in spheroid size when compared with nanoparticle treatment alone, apart from spheroids exposed to Fe_3_O_4_@PEG 6K (Figure 5a–h). In this case, irradiation resulted in a modest but statistically significant reduction in the mean spheroid diameter of approximately 0.15 mm (*p* = 0.01). Despite the absence of pronounced dimensional changes, morphological alterations were observed following exposure to nanoparticles and/or ionizing radiation. Specifically, melanoma spheroids treated with Fe_3_O_4_@PEG 6K exhibited partial disintegration in the peripheral regions (Figure 5c,d), while spheroids treated with Gd-doped nanoparticles additionally showed structural disruption within the central regions (Figure 5e–h).

Cell viability in 3D melanoma spheroid models was assessed using the MTS tetrazolium salt assay (Figure 5i,k). In the model containing a lower proportion of melanoma cells (2500 B16 + 5000 L929, Figure 5i), the results showed that the dual treatment (nanoparticles followed by irradiation) induced a significant decrease in cell viability compared with spheroids exposed only to nanoparticles alone, indicating a cytotoxic effect in the case of Fe_3_O_4_@PEG 6K (*p* < 0.01). Apart from this condition, the MTS assay did not reveal other statistically significant cytotoxic effects following the dual treatment in the 3D spheroids.

Mitochondrial metabolism, evaluated using the ATP bioluminescence assay (Figure 5j,l), was significantly affected by the dual treatment compared with both nanoparticle-only treatment and the negative control for all nanoparticle types in the model with a lower melanoma cell content (2500 B16 + 5000 L929) (Figure 5l). The observed reduction in ATP levels indicates inhibition of the energetic metabolism of melanoma cells. In the spheroid model with a higher melanoma cell proportion (5000 B16 + 2500 L929), mitochondrial metabolism was reduced to levels considered cytotoxic in samples exposed to all nanoparticle formulations. This effect was statistically significant following the combined treatment when compared with both the negative control and the nanoparticle-only samples (Figure 5l). Overall, these results support the potential of the investigated Fe-Gd-based nanoparticles as effective radiosensitizing agents in 3D melanoma models.

To further validate the statistical relevance of these observations, an extended statistical analysis was performed using one-way ANOVA followed by Tukey’s multiple comparison test and two-way ANOVA. In the model containing a lower proportion of melanoma cells (2500 B16 + 5000 L929), two-way ANOVA revealed significant effects of nanoparticle exposure (*p* < 0.001) and the radiation dose (*p* = 0.015), as well as a significant interaction between nanoparticle treatment and irradiation (*p* = 0.0108), indicating that the cellular response to irradiation depended on nanoparticle exposure. One-way ANOVA confirmed significant differences among treatment groups (F = 5.87, *p* < 0.001), with combined nanoparticle–irradiation treatments producing lower viability compared with irradiation alone. ATP measurements in the same model further showed significant effects of nanoparticle treatment and irradiation (both *p* < 0.001), together with a significant interaction between these factors (*p* < 0.001), indicating pronounced impairment of mitochondrial metabolism under dual treatment conditions. In contrast, in the model containing a higher proportion of melanoma cells (5000 B16 + 2500 L929), two-way ANOVA indicated that nanoparticle exposure significantly affected spheroid viability (*p* < 0.001), whereas irradiation alone did not significantly influence metabolic activity (*p* = 0.664), and no significant interaction between nanoparticle treatment and irradiation was detected (*p* = 0.232). One-way ANOVA confirmed significant differences among treatment groups (F = 8.99, *p* < 0.001), with the 15% Gd formulation producing the most pronounced reduction in viability. For ATP levels, both nanoparticle treatment (*p* = 0.0014) and irradiation (*p* < 0.001) significantly influenced mitochondrial metabolism, although no interaction between the two factors was observed (*p* = 0.439), suggesting that energetic alterations in this model were primarily driven by nanoparticle exposure and irradiation independently.

Taken together, the results obtained on 2D cellular models demonstrated that Fe and/or Gd oxide nanoparticles were generally biocompatible in the absence of ionizing radiation and exhibited biological effects predominantly under ionizing radiation conditions. While metabolic and mitochondrial assays revealed the adaptive response of normal fibroblasts, which include preserved energetic production and increased mitochondrial mass following irradiation, melanoma cells exhibited a distinct vulnerability, which was characterized by an impaired energetic metabolism and a reduced long-term proliferative capacity. These findings indicated that the radiosensitizing effect of the investigated nanoparticle formulations was not determined by an acute cytotoxic effect, but rather by a modulatory stress reaction of the cells, occurring at the mitochondrial level, which appeared to be distinctly regulated in normal versus tumor cells.

This interpretation was further supported by the clonogenic assay, which ultimately reflected the cell division incapacity. Although irradiation alone induced a significant reduction in colony formation in both normal fibroblasts and melanoma cells, the absence of a pronounced differential effect should be interpreted in the context of a relatively high single radiation dose. It is important to note that the reduction in clonogenic survival in normal fibroblast cells was not accompanied by severe metabolic dysfunction, suggesting that the activation of compensatory mechanisms preserved the cellular homeostasis despite the impaired long-term division capacity. In contrast, nanoparticle-mediated radiosensitization was highlighted in melanoma cells and associated with concomitant alterations of the metabolic activity, the mitochondrial function, and clonogenic survival, which might indicate a reduced ability to combine oxidative and radiative stress adaptation [67].

The relevance of these observations was further reinforced by the results obtained in 3D melanoma spheroid models, which more closely simulated the architectural, diffusion, and metabolic constraints of solid tumors. In these models, the combined nanoparticle-irradiation treatment consistently impaired mitochondrial metabolism, even in the absence of major changes in the spheroid size, highlighting the sensitivity of energetic pathways as an early indicator of treatment efficiency. The ability of Fe and Fe-Gd oxide nanoparticles to potentiate the radiation-induced metabolic disruption in 3D systems showcased their translational potential and supported the hypothesis that the radionsesitization effect might become more pronounced in complex biological environments. These findings provided a strong rationale for extending the investigations towards in vivo models, particularly to assess the systemic tolerance and response in non-tumoral tissues.

In the context of the existing literature, most studies on Gd-based radiosensitizers are based on molecular systems such as gadolinium chelates, where the radiosensitizing effect is primarily attributed to high-Z dose enhancement mechanisms. Clinically translated platforms such as AGuIX^®^ exemplify this approach, focusing on ultrasmall chelated Gd systems with favorable biodistribution and radiotherapy enhancement [77]. Subsequent developments have further functionalized these systems, for example through terbium doping to enhance ROS-mediated effect therapy [78] or by combining multiple high-Z elements, such as Au-Gd nanosystems [79], to improve therapeutic efficiency. In parallel, other strategies have explored Gd-based oxide nanoparticles or hybrid nanostructures designed for multimodal therapy, often integrating targeting ligands or additional therapeutic components [80].

In contrast, Fe-based radiosensitizers are generally limited by the lower atomic number of iron, although they benefit from high biocompatibility and efficient cellular internalization. To overcome this limitation, several studies have proposed composite or hybrid Fe-Gd systems, typically involving surface functionalization [81], the incorporation into mesoporous carriers [82,83], or combinations with other materials for imaging or therapeutic purposes. However, in most of these cases, Gd is either present as a separate phase [81,84], as a surface-bound species [77,78,79,80], or within complex hybrid architectures [82,83], rather than being structurally integrated into the iron oxide lattice.

Approaches involving Gd incorporation into the Fe_3_O_4_ crystalline lattice have been primarily investigated in the context of the magnetic properties [85,86,87], imaging performance [86,88], or hyperthermia applications [88,89], with limited focus on radiosensitization and without a systematic evaluation of composition-dependent biological effects [90].

In this context, the present study provides a distinct approach by investigating a controlled series of Fe-Gd oxide nanoparticles in which Gd is incorporated within the iron oxide structure through partial ionic substitution. By systematically varying the Gd content and correlating structural modifications with biological responses, this work demonstrates that radiosensitization is not only governed by the elemental composition, but rather by the interplay between crystallinity, phase composition, and cellular metabolic adaptability. Importantly, the integration of metabolic (MTT/MTS), mitochondrial (ATP, MitoTracker), and clonogenic endpoints in both 2D and 3D melanoma models enables the identification of a composition-dependent therapeutic window, highlighting the 25% Gd formulation as an optimal balance between structural integrity and functional radiosensitization. This multi-level evaluation provides a more comprehensive functional framework compared to conventional studies, which typically assess isolated endpoints or single nanoparticle formulations.

### 3.5. In Vivo Evaluation of Systemic Tolerance and Tissue Response

Iron oxide nanoparticles doped with 25% Gd were selected for an in vivo tolerance evaluation in non-tumoral models, as these nanoparticles demonstrated the most favorable performance in the in vitro 3D melanoma models. Following the intraperitoneal administration of nanoparticle suspensions prepared in physiological saline at concentrations consistent with previous experiments on PEG-coated iron oxide nanoparticles [91], their systemic tolerance and tissue response was assessed in BALB/c mice. The presence of nanoparticles in major organs was analyzed at different time points, from 24 h up to 7 days post-injection, allowing the evaluation of potential effects on healthy tissues and providing insight into their in vivo clearance behavior. Tissue samples were collected from organs directly involved in systemic circulation and physiological elimination pathways, which are known to represent key sites for nanoparticle accumulation and clearance [92,93].

A histopathological examination of the collected tissue samples revealed no detectable accumulation of nanoparticles in the liver, kidneys, lungs, myocardium, intestine, or brain at any of the investigated time points (Supplementary Materials, Figures S13–S16B–G). Moreover, no histopathological alterations were observed in these organs, indicating the absence of detectable tissue damage following nanoparticle administration. In contrast, the presence of nanoparticles was consistently observed in spleen tissue at all analyzed time intervals, appearing as bright blue aggregates (Supplementary Materials, Figures S13–S16A). A marked increase in nanoparticle accumulation was noted at later time points, particularly at 7 days post-administration (Supplementary Materials, Figure S16A).

A detailed analysis of spleen sections showed that most nanoparticle aggregates were localized within the red pulp, specifically at the level of the splenic Billroth cord network and in close proximity to the splenic sinuses (Supplementary Materials, Figures S11–S16A). These regions are involved in blood filtration, suggesting that the spleen plays a major role in the sequestration and elimination of Gd-doped iron oxide nanoparticles. This observation is consistent with our previous reports regarding the biosafety profile and clearance pathways of iron oxide nanoparticles [94,95]. In addition, a limited number of nanoparticle aggregates were identified within macrophages located in the marginal zones of the white pulp (Supplementary Materials, Figures S11–S16A), suggesting that these nanoparticles might undergo opsonization and subsequent phagocytic uptake [96,97]. Similar findings regarding spleen accumulation of iron oxide-based nanoparticles as early as the first day after systemic administration have been reported in other studies [98,99].

Overall, the in vivo findings indicated a favorable tolerance profile of the 25% Gd-doped iron oxide nanoparticles following systemic administration, with no detectable accumulation or histopathological alterations in major organs, except for spleen-associated sequestration consistent with the physiological clearance mechanisms. These observations were in line with the in vitro results, which demonstrated that the biological effects of Fe and Fe-Gd oxide nanoparticles were not driven by intrinsic cytotoxicity but were context-dependent. Together, the in vitro and in vivo data support the concept that the radiosensitizing activity of the investigated nanoparticles occurs under specific biological conditions, while maintaining acceptable systemic tolerance, thereby justifying further investigation in tumor-bearing in vivo models.

## Data Availability

The original contributions presented in this study are included in the article. Further inquiries can be directed at the corresponding authors.

## Supplementary Materials

The following supporting information can be downloaded at: https://www.mdpi.com/article/10.3390/pharmaceutics18050525/s1.
