# Supplementary material for: Gadolinium-Doped Iron Oxide Nanoparticles Enhance Radiosensitivity in Melanoma Models Associated with Metabolic Dysfunction
Source: Pharmaceutics. 2026 Apr 26;18(5):525. doi: 10.3390/pharmaceutics18050525 (PMC13210800; doi:10.3390/pharmaceutics18050525)
Supplement: Supplementary file 1 [file pharmaceutics-18-00525-s001.zip › pharmaceutics-4241524-supplementary.pdf]

Supplementary Materials. Gadolinium-Doped Iron Oxide Nanoparticles Enhance Radio-sensitivity in Melanoma Models Associated with Metabolic Dysfunction

S1. Scanning Electron Microscopy (SEM)

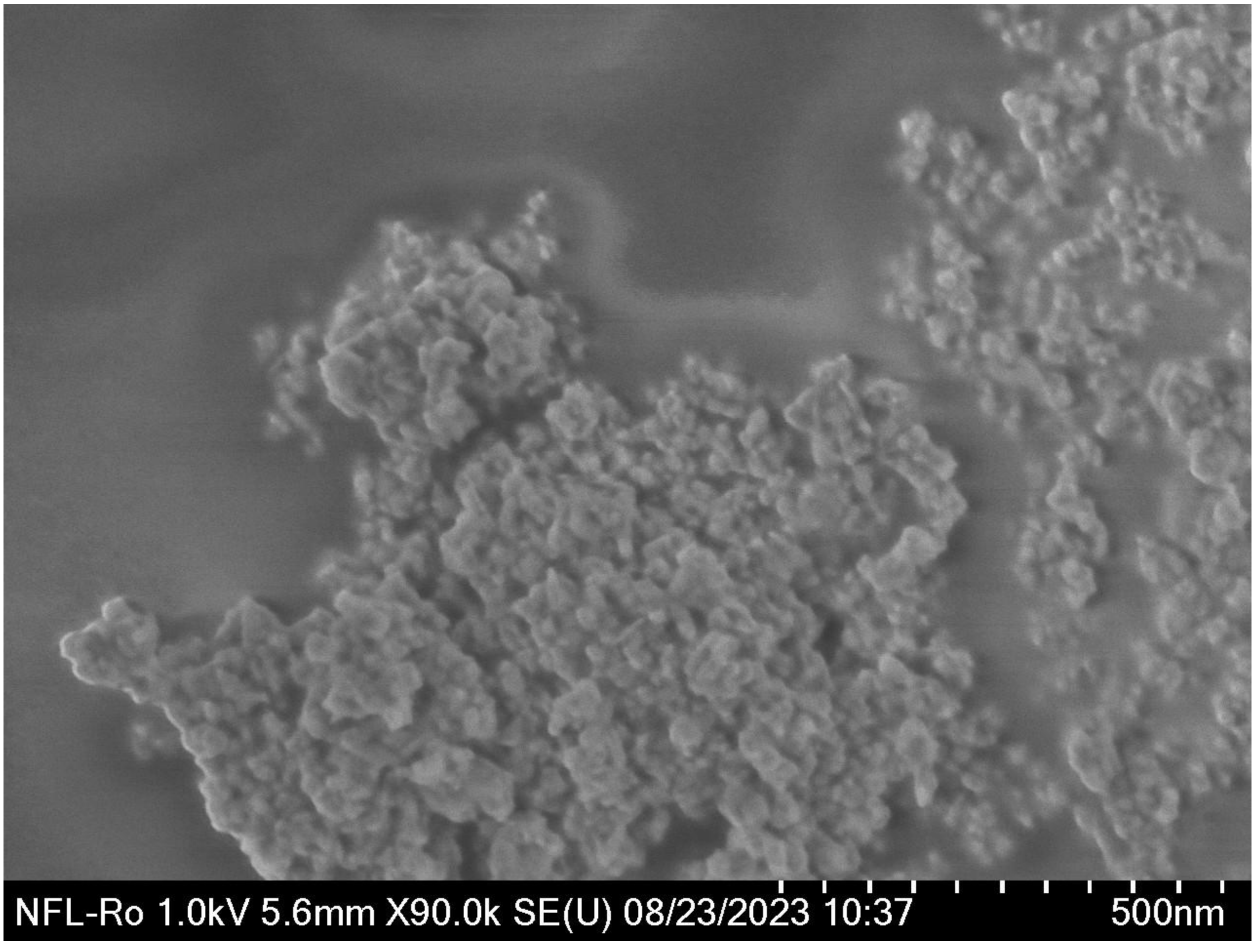

Figure S1- Scanning Electron Microscopy (SEM) of Fe_3O_4@PEG 6K nanoparticles

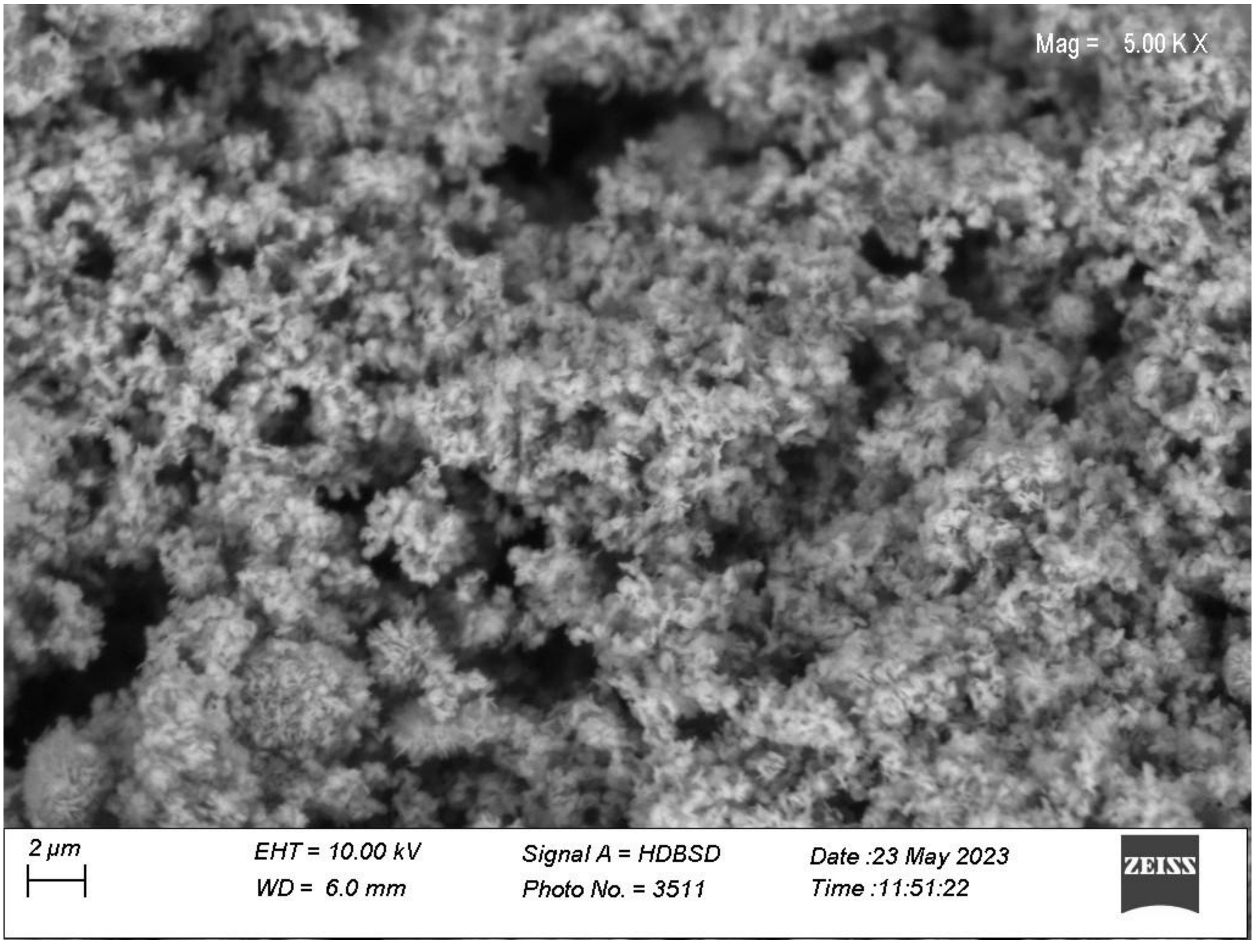

Figure S2- Scanning Electron Microscopy (SEM) of Gd_2O_3@PEG 6K nanoparticles

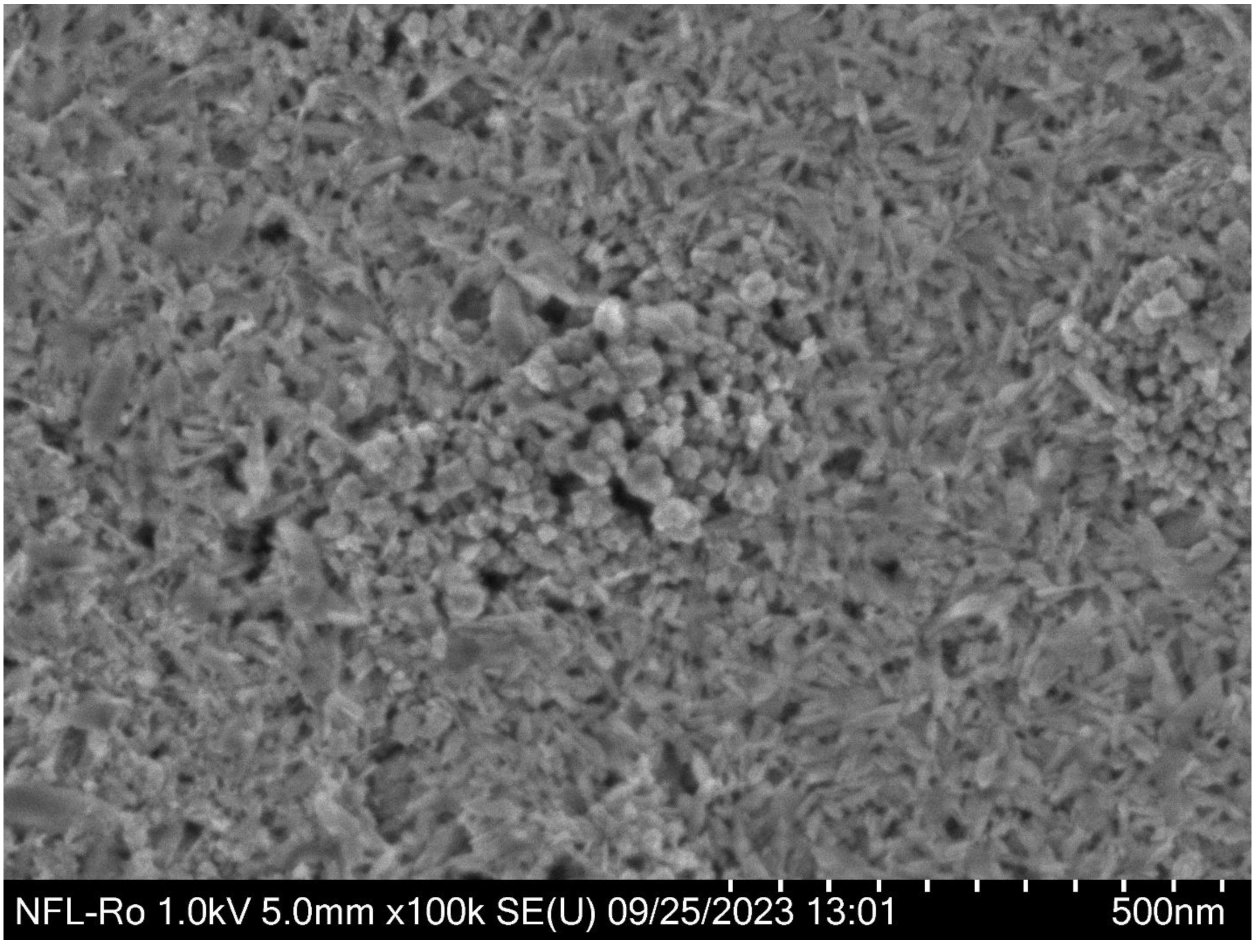

Figure S3- Scanning Electron Microscopy (SEM) of 10% Gd^{3+}@PEG 6K nanoparticles

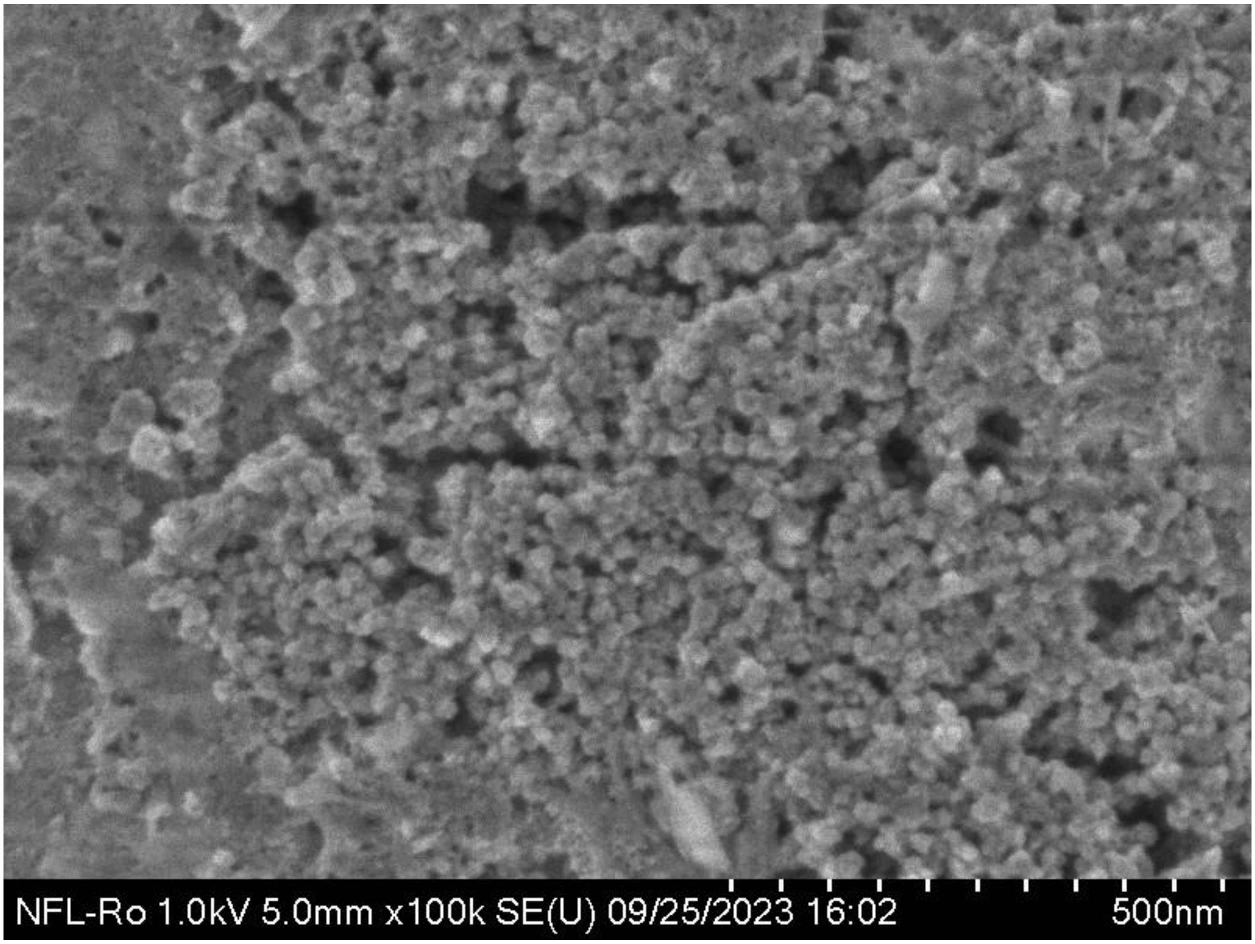

Figure S4- Scanning Electron Microscopy (SEM) of 15% Gd^{3+}@PEG 6K nanoparticles

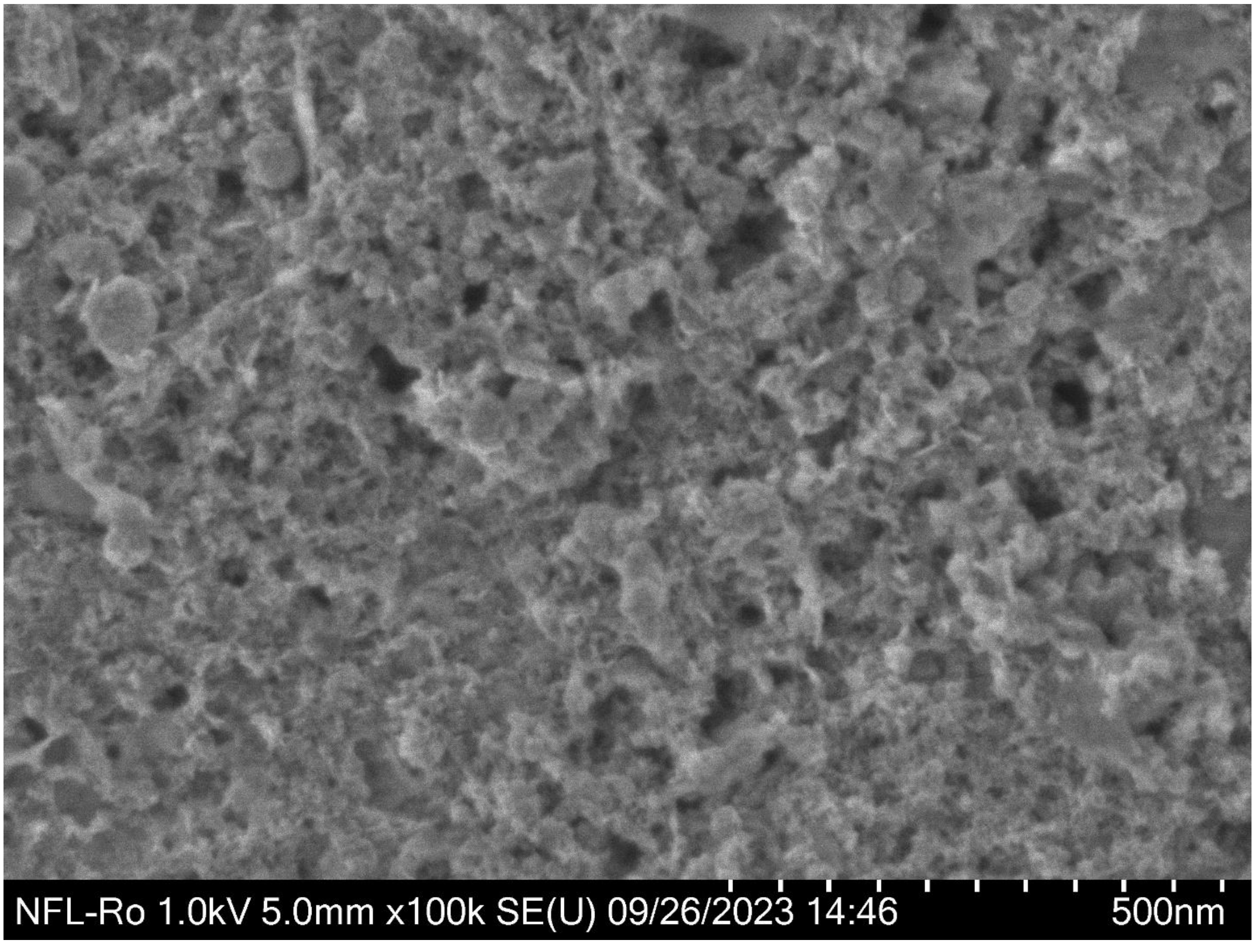

Figure S5- Scanning Electron Microscopy (SEM) of 25% Gd^{3+}@PEG 6K nanoparticles

S2. X-Ray Diffraction analysis (XRD)

Table S1. Diffraction interferences identified for Fe and Gd oxide nanoparticles.

Sample	Peak position (degrees)	Interplanar distance	Crystalline plane (hkl)	Crystallite dimension (Å)
Fe_3O_4@PEG 6K	30.21	2.9558	(220)	91
	35.58	2.5209	(311)	93
	43.17	2.0936	(400)	124
	53.49	1.7117	(422)	130
	57.25	1.6080	(511)	84
	62.75	1.4794	(440)	91
Gd_2O_3@PEG	26.23	3.3937	/(101)	975
	26.75	3.3292	/(002)	535

	28.57	3.1214	(222)	218
	32.05	2.7901	(111)	827
	35.23	2.5453	/(102)	530
	40.60	2.2198	(422)	538
	42.21	2.1391	(112)	524
	45.95	1.9735	(521)/ (200)	621
	48.02	1.8929	(433)/ (201)	663
	52.6	1.7382	(611)/ (113)	481
	53.66	1.7065	(026)/(211)	514
	59.16	1.5602	/(212)	533
	60.23	1.5352	(543)	440
	62.92	1.4758	(633)	630
	65.14	1.4309	/(114)	411
	66.98	1.3959	(237)/ (220)	513
	73.32	1.2902	(822)	213
	76.165	1.2488	(662)	484
	77.97	1.2243	(311)	368
	78.92	1.2120	(048)	311
10% Gd^{3+}	30.21	2.9559	(220)	131
	35.55	2.5230	(311)	88
	43.4	2.0845	(400)	58
	53.7	1.7068	(422)	38
	57.23	1.6083	(511)	94
	62.81	1.4782	(440)	88
15% Gd^{3+}	30.25	2.9517	(220)	179
	35.53	2.5244	(311)	109
	43.25	2.0903	(400)	180
	53.68	1.7060	(422)	155
	57.22	1.6008	(511)	99
	62.83	1.4778	(440)	110
25%	30.24, 30.32	2.9526	(220)	380, 381

	35.57, 35.66	2.5217	(311)	157, 158
	43.32, 43.43	2.0869	(400)	378, 380
	57.20, 57.35	1.6092	(422)	239, 240
	62.76, 62.93	1.4793	(511)	129, 130

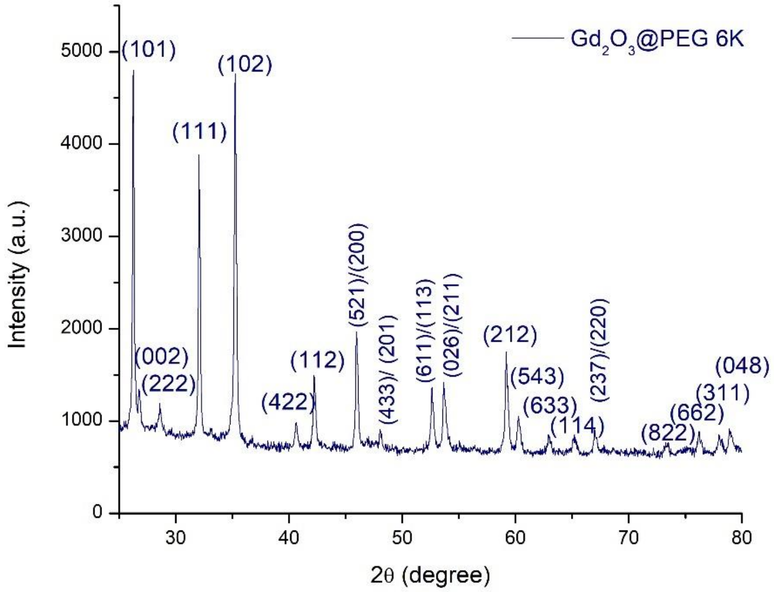

Figure S6. X-Ray Diffraction interferences for Gd oxide nanoparticles.

S3. Energy Dispersive X-Ray Spectroscopy (EDX)

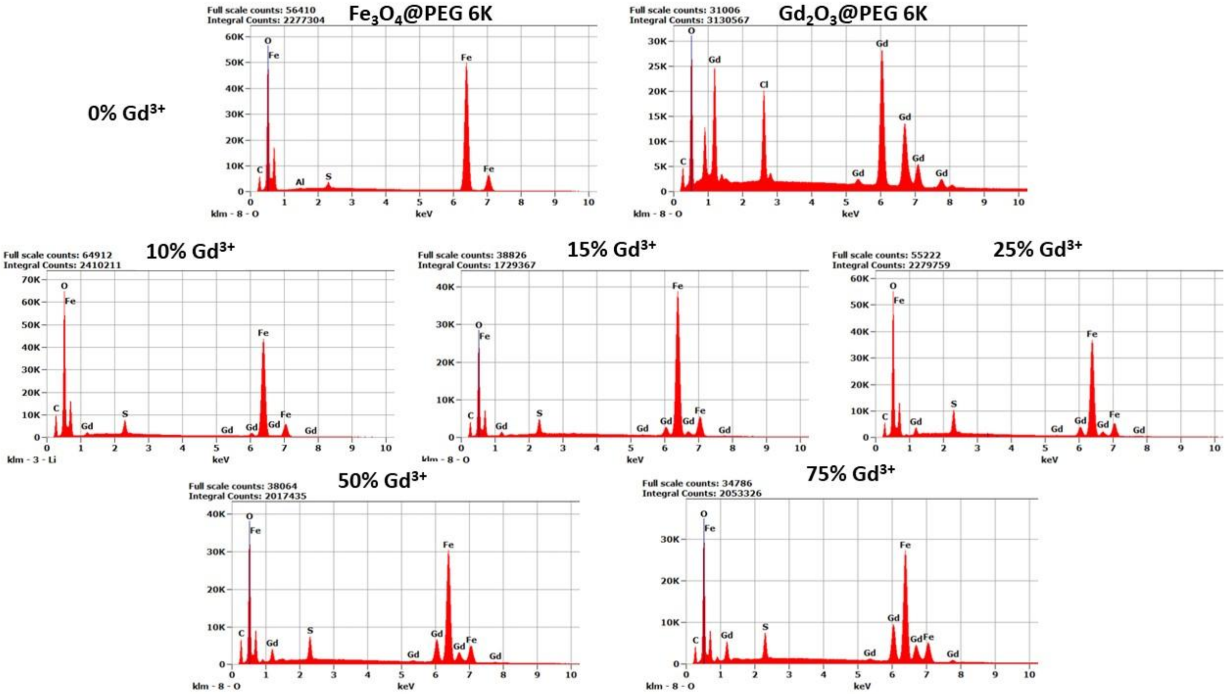

Figure S7. Energy Dispersive X-Ray Spectroscopy spectrums for Fe and/or Gd oxide nanoparticles

Table S2. shows the elemental composition (in weight percent) of the Fe and/or Gd oxide samples obtained from the syntheses. The Gd content added during the precipitation reaction was slightly lower (by approximately 2–3 wt%) than the values measured by EDX analysis, suggesting that the precipitation of Fe^{2+} and Fe^{3+} ions did not proceed with full efficiency. In contrast, the overall yield of the composite was maximal, except for the 10% Gd^{3+} sample.

Table S2. Elemental composition (in weight percent) of the Fe and/or Gd oxide samples obtained.

Sample	Added wt% Gd	Elemental composition (wt%)			
		Fe	Gd	O	Impurities (including C)
Fe_3O_4@PEG 6K	0	62.04±0.14	0	26.83±0.09	11.13±0.1
Gd_2O_3@PEG 6K	60	0	67.45±0.22	18.44±0.08	14.1±0.12
10% Gd^{3+}	3.22	47.81±0.13	2.77±0.14	31.78±0.11	17.64±0.09
15% Gd^{3+}	4.83	60.72±0.18	7.74±0.2	19.12±0.08	12.42±0.1
25% Gd^{3+}	8.06	46.94±0.15	10.04±0.18	29.59±0.1	13.43±0.09
50% Gd^{3+}	16.13	40.6±0.15	19.05±0.21	24.37±0.1	15.97±0.09

75% Gd^{3+}	24.19	37.00±0.16	28.99±0.24	22.44±0.09	11.56±0.1

S4. Morphological assessment of the 3D melanoma cell model

S4.1 First model: seeding of L929 fibroblast cells followed by the addition of B16 melanoma cells

The liquid overlay technique was employed to obtain a 3D melanoma cell model, enabling the formation of morphologically homogenous spheroids. For this purpose, non-adherent spheroid plates (Corning, New York, USA) were used, and a monocellular suspension of L929 fibroblast cells was mixed with murine Collagen type I (Sigma Aldrich, Darmstadt, Germany) at different concentrations. Following incubation under standard cell culture conditions, fibroblast spheroids were formed, and B16 melanoma cells were subsequently added at different concentrations on top of the pre-formed spheroids. This two-step approach was designed to provide an anchoring structure for the melanoma cells and to promote spheroid formation.

Step 1 consisted of L929 cell seeding and spheroid generation. For the first step, L929 cells were detached from culture flasks by trypsinization and subsequently counted. Six cell suspensions were prepared, with concentrations of 5000 cells/200 μL and 2500 cells/200 μL per well (three suspensions of each concentration). Murine type I collagen was then added at final volumes of 0.1, 0.05, and 0.025 μL. The fibroblast cells suspensions were gently mixed and 200 μL of each suspension was added into individual wells of the spheroid culture plates. Cells were incubated for 72h under standard conditions to allow spheroid formation. The experimental conditions used in this first step are summarized in Figure S8 a and b.

After incubation, relatively large spheroids were obtained. Brightfield images of the resulting spheroids were acquired using an optical microscope at 5x magnification (Figure S8 c). It was observed that the use of low collagen concentrations combined with lower L929 cells densities led to the formation of fusiform-shaped spheroids with compact structures, a morphology considered favorable for the development of 3D cell culture models.

The mean diameter of the resulting spheroids was measured using the ImageJ software (National Institutes of Health, Bethesda, MA, USA), based on an image of a 1 mm calibration ruler acquired at 5x magnification. Subsequently, spheroid images were converted into binary format to evaluate the perimeter, surface area, and shape descriptor parameters, including aspect ratio (AR), roundness, circularity, and solidity, using the particle analysis function implemented in the software. The results were obtained from three independent experiments, each performed in triplicate, and the data were expressed as mean ± standard deviation (Table S3).

After three days of incubation, L929 spheroids (Table S3) with a mean diameter ranging from 0.46 to 0.51 mm were obtained, exhibiting an elongated morphology, as indicated by aspect ratio values between 1.17 and 1.30. The spheroids showed relatively low circularity values (approximately

0.3), while roundness values were closer to the reference value of 1 (approximately 0.80-0.85). The solidity of the fibroblast spheroids was relatively high (approximately 0.9), indicating compact structures and supporting their suitability for use in the development of a 3D melanoma cell model.

Step 2 consisted of detaching B16 melanoma cells from culture flasks by trypsinization, followed by cell counting. Cell suspensions were prepared, with concentrations of 5000 cells/100 μL and 2500 cells/100 μL of cell culture medium (three suspensions for each concentration). Murine type I collagen was subsequently added at final volumes of 0.05, 0.025, and 0.0125 μL. 100 μL of the culture medium in the wells containing the pre-formed L929 spheroids was then replaced with 100 μL of the B16 cell suspension, thereby bringing the B16 cells into direct contact with the previously generated L929 spheroids. Cells were incubated for an additional 72 h under standard conditions to allow the formation of the 3D melanoma cell model. The experimental conditions employed in this step are summarized in Figure S8d and e. Brightfield images of the resulting 3D melanoma cell models were acquired using an optical microscope at 5x magnification (Figure S8 f). The obtained spheroids exhibited relatively compact structures with irregular morphology.

The mean diameter of the resulting spheroids was measured using the ImageJ software (National Institutes of Health, Bethesda, MA, USA), based on an image of a 1 mm calibration ruler acquired at 5x magnification. Subsequently, spheroid images were converted into binary format to evaluate the perimeter, surface area, and shape descriptor parameters, including aspect ratio (AR), roundness, circularity, and solidity, using the particle analysis function implemented in the software. The results were obtained from three independent experiments, each performed in triplicate, and the data were expressed as mean ± standard deviation (Table S4).

The morphological assessment of the L929-B16 spheroids obtained using the two-step approach was performed after 72 h of incubation under standard cell culture conditions following the addition of melanoma cells. At this timepoint, the resulting spheroids exhibited highly non-homogeneous morphology, with mean diameters ranging from 0.77 to 1.04 mm. The spheroids showed very low circularity values (0.18-0.45) but relatively high solidity (0.74-0.90). Roundness values were relatively close to the reference value of 1 (0.72-0.82), except for the model with the highest cell density (5000 L929 + 5000 B16) combined with the lowest collagen concentration. This observation highlights the importance of extracellular matrix components in the experimental design.

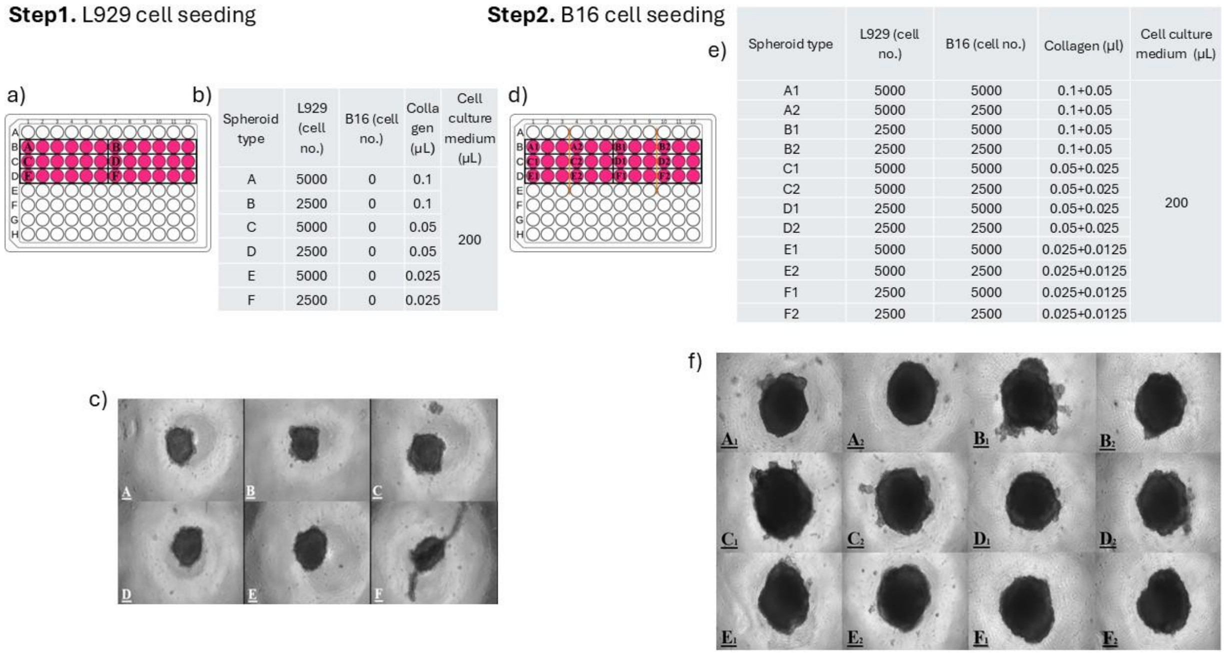

Figure S8. Generation of the 3D melanoma cell models using the two-step approach: a) experimental design and (b) experimental conditions used in the first step of seeding the L929 fibroblast cells, (c) brightfield images of L929 spheroids generated after 72h of incubation in standard cell culture conditions, corresponding to each experimental condition; (d) experimental design and (e) experimental conditions used in the second step of seeding B16 melanoma cells on top of the previously generated fibroblast spheroids, (f) brightfield images of 3D melanoma cells models generated after another 72h of incubation in standard cell culture conditions, corresponding to each experimental condition;

Table S3. Morphological parameters of L929 spheroids after 72h of incubation.

Spheroid Type				Diameter (mm)	Surface (mm^2)	Perimeter (mm)	Circularity	AR	Roundness	Solidity
	L929 (cell no.)	B16 (cell no.)	Collagen (µL)							
A	5000	0	0.1	0.5±0.05	0.18±0.01	2.65±0.33	0.32±0.06	1.24±0.02	0.8±0.01	0.88±0.03
B	2500	0	0.1	0.46±0.03	0.15±0.01	2.63±0.33	0.29±0.07	1.17±0.08	0.85±0.05	0.87±0.01
C	5000	0	0.05	0.51±0.05	0.19±0.02	2.99±0.46	0.29±0.07	1.3±0.22	0.78±0.12	0.88±0.3
D	2500	0	0.05	0.46±0.02	0.14±0.008	2.6±0.12	0.29±0.04	1.26±0.17	-	0.88±0.01
E	5000	0	0.025	0.48±0.02	0.18±0.01	2.82±0.23	0.29±0.04	1.24±0.13	0.81±0.08	0.89±0.01

F	2500	0	0.025	-	-	-	-	-	-	-

Table S4. Morphological parameters of L929-B16 spheroids generated using the two-step approach after 72h of incubation.

Spheroid Type				Diameter (mm)	Surface (mm^2)	Perimeter (mm)	Circularity	AR	Roundness	Solidity
	L929 (cell no.)	B16 (cell no.)	Collagen (µL)							
A1	5000	5000	0.1+0.05	0.79±0.06	0.43±0.03	3.87±0.76	0.39±0.14	1.25±0.2	0.81±0.13	0.88±0.03
A2	5000	2500		0.77±0.06	0.46±0.07	3.68±0.79	0.45±0.13	1.224±0.04	0.8±0.03	0.93±0.01
B1	2500	5000		1.04±0.33	0.79±0.27	8.84±3.56	0.14±0.07	1.45±0.44	0.72±0.19	0.79±0.03
B2	2500	2500		-	-	-	-	-	-	-
C1	5000	5000	0.05+0.025	-	-	-	-	-	-	-
C2	5000	2500		0.82±0.05	1.02±0.53	6.95±3.26	0.32±0.19	1.37±0.24	0.74±0.13	0.74±0.17
D1	2500	5000		0.87±0.72	0.68±0.5	8.59±6.73	0.18±0.14	1.24±0.23	0.82±0.14	0.77±0.13
D2	2500	2500		-	-	-	-	-	-	-

E1	5000	5000	0.025+0.0125	0.93±0.18	0.42±0.4	3.96±1.76	0.26±0.21	3.1±0.50	0.42±0.37	0.9±0.03
E2	5000	2500		0.82±0.11	0.71±0.5	5.95±4.55	0.36±0.21	1.27±0.1	0.78±0.06	0.84±0.18
F1	2500	5000		0.86±0.09	0.74±0.48	6.36±3.26	0.29±0.21	1.26±0.12	0.79±0.07	0.81±0.15
F2	2500	2500		0.86±0.13	0.47±0.07	4.24±0.87	0.35±0.11	1.42±0.35	0.72±0.15	0.88±0.06

S4.2 Second model: simultaneous seeding of L929 fibroblast cells and B16 melanoma cells

The liquid overlay technique was employed to obtain a 3D melanoma cell model, enabling the formation of morphologically homogenous spheroids. For this purpose, non-adherent spheroid plates (Corning, New York, USA) were used, and a multicellular suspension of L929 fibroblast cells and B16 melanoma cells was mixed with murine Collagen type I (Sigma Aldrich, Darmstadt, Germany) at different concentrations. Following incubation under standard cell culture conditions, spheroids were formed.

L929 cells and B16 cells were detached from culture flasks by trypsinization and subsequently counted. Multicellular cell suspensions were prepared in complete culture medium: 2500 B16 cells+2500 L929 cells/200 μL, 2500 B16 cells + 5000 L929 cells/ 200μL, 5000 B16 cells + 2500 L929 cells/ 200μL, 5000 B16 cells+ 5000 L929 cells/ 200μL, as well as monocellular cell suspensions 2500/5000 B16 cells, 2500/5000 L929 cells. Murine type I collagen was then added at final volumes of 0.05 μL. The cells suspensions were gently mixed and 200 μL of each suspension was added into individual wells of the spheroid culture plates. Cells were incubated for 72h under standard conditions to allow spheroid formation. The experimental conditions used in this first step are summarized in Figure S9 a and b.

After incubation, relatively large spheroids were obtained. Brightfield images of the resulting spheroids were acquired using an optical microscope at 5x magnification (Figure S9 c). The mean diameter of the resulting spheroids was measured using the ImageJ software (National Institutes of Health, Bethesda, MA, USA), based on an image of a 1 mm calibration ruler acquired at 5x magnification. Subsequently, spheroid images were converted into binary format to evaluate the perimeter, surface area, and shape descriptor parameters, including aspect ratio (AR), roundness, circularity, and solidity, using the particle analysis function implemented in the software. The

results were obtained from three independent experiments, each performed in triplicate, and the data were expressed as mean ± standard deviation (Table S5).

The morphological assessment of the L929-B16 spheroids obtained using the one-step approach was performed after 72 h of incubation under standard cell culture conditions. At this timepoint, the resulting spheroids with non-equal cell concentrations exhibited relatively homogeneous morphology, with mean diameters of about 0.64 mm. The spheroids showed relatively low circularity values (0.36-0.37) but relatively high solidity (0.89-0.90). Roundness values were relatively close to the reference value of 1 (0.76-0.8). Given these good characteristics, the models containing unequal quantities of cells were selected for further phenotypic characterization and nanoparticle investigations.

a)

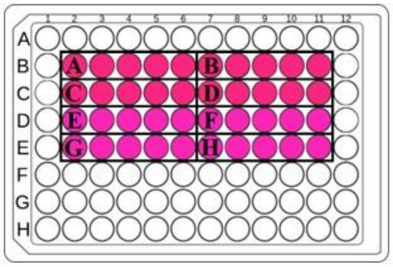

b)

Spheroid type	L929 (cell no.)	B16 (cell no.)	Collagen (µL)	Cell culture medium (µl)
A	2500	0		
B	0	2500		
C	5000	0		
D	0	5000		
E	5000	5000	0.05	200
F	5000	2500		
G	2500	5000		
H	2500	2500		

c)

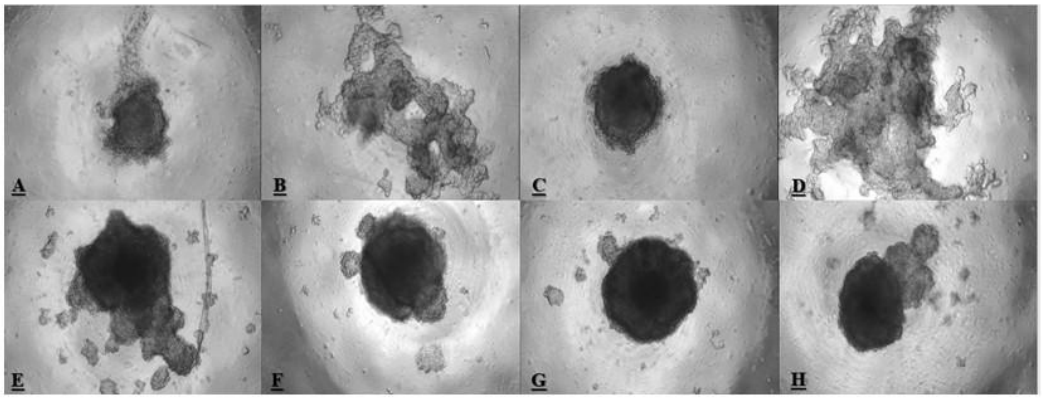

Figure S9. Generation of the 3D melanoma cell models using the one approach: a) experimental design and (b) experimental conditions used for the seeding of L929 fibroblast and melanoma cells, (c) brightfield images of 3D melanoma cells models generated after another 72h of incubation in standard cell culture conditions, corresponding to each experimental condition;

Table S5. Morphological parameters of L929-B16 spheroids generated using the one-step approach after 72h of incubation.

Spheroid Type				Circularity	AR	Roundness	Solidity

	L929 (cell no.)	B16 (cell no.)	Collagen (µL)	Diameter (mm)	Surface (mm^2)	Perimeter (mm)				
A	2500	0	0.05	0.45 ± 0.04	0.16 ± 0.02	3.13 ± 0.82	0.22 ± 0.09	1.33±0.16	0.76 ± 0.08	0.85 ± 0.04
B	0	2500	0.05	-	-	-	-	-	-	-
C	5000	0	0.05	0.5 ± 0.05	0.19 ± 0.01	3.29 ± 0.2	0.23 ± 0.03	1.18±0.08	0.85 ± 0.06	0.87 ±0.02
D	0	5000	0.05	-	-	-	-	-	-	-
E	5000	5000	0.05	0.71 ± 0.08	0.37 ± 0.05	4.69 ± 0.86	0.22 ± 0.06	1.54±0.13	0.65 ± 0.05	0.82 ± 0.06
F	5000	2500	0.05	0.64 ± 0.04	0.32 ± 0.03	3.42 ± 0.62	0.36 ± 0.11	1.34±0.15	0.76 ± 0.08	0.89 ± 0.04
G	2500	5000	0.05	0.64 ± 0.03	0.34 ± 0.03	3.51 ± 0.7	0.37 ± 0.13	1.27±0.18	0.8 ± 0.11	0.9 ± 0.05
H	2500	2500	0.05	0.62 ± 0.08	0.29 ± 0.04	3.36 ± 0.44	0.33 ± 0.06	1.48±0.29	0.7 ± 0.14	0.85 ± 0.04

S4.3 Immunocytochemical characterization of the 3D multi-cellular models

For the immunocytochemical characterization of the 3D melanoma models, spheroids were processed according to a standardized histological protocol. Prior to harvesting, spheroids were incubated for 2 h with 30 µM bromodeoxyuridine (BrdU), a thymidine analogue incorporated into the DNA of proliferating cells, and with 300 µM pimonidazole (Pimo), a nitroimidazole compound used as a marker of tissue hypoxia.

Following incubation, spheroids were collected in microcentrifuge tubes, the supernatant was carefully removed, and samples were washed several times with PBS. Fixation was performed overnight at 4 °C using 4% Roti-Histofix (Carl Roth, Karlsruhe, Germany). Subsequently, spheroids were dehydrated by immersion in 50% and 70% ethanol solutions (15 min each), embedded in Histogel (Thermo Fisher Scientific, Waltham, MA, USA), allowed to solidify in plastic molds, and transferred into histocassettes.

Further dehydration was carried out by successive immersion in ethanol solutions of increasing concentration (80-100%) for 30-45 min each, followed by two immersions in 100% xylene (45 min each). Samples were then infiltrated with molten paraffin through three successive incubations (45 min each) and embedded into paraffin blocks, which were allowed to harden. Embedded samples were stored overnight at -20 °C prior to sectioning. Serial sections of 6 µm thickness were obtained using a microtome, mounted onto Superfrost glass microscope slides (Thermo Fisher Scientific, Waltham, MA, USA), and dried overnight at 37 °C.

Paraffin sections were subjected to deparaffinization and rehydration by immersion in 100% xylene (three times, 10 min each), followed by graded ethanol solutions (100-40%), and finally rinsed in deionized water. Antigen retrieval was performed by heating the sections in citrate buffer for 20 min at 600 W using a microwave oven. After cooling on ice for 30 min, slides were washed

several times with Tris-buffered saline (TBS). Endogenous peroxidase activity was quenched by incubation in 3% hydrogen peroxide in methanol for 10 min, followed by additional TBS washes.

Immunostaining for BrdU and Pimo was performed using anti-BrdU rat monoclonal antibody (ab6326, Abcam, Cambridge, UK) and Hypoxyprobe-1 Omni kit PAb 2627(AP) Rabbit antisera (Hypoxyprobe, Burlington, MA, USA) respectively. Non-specific binding was blocked by incubation with 1-5% normal serum in primary antibody dilution buffer for 60 min at room temperature. Primary antibodies were diluted according to the manufacturer's instructions and incubated with the sections overnight at 4 °C. Immunostaining for Melan-A (anti-MelanA rabbit monoclonal antibody, ab51061, Abcam, Cambridge, UK) and S100 (anti-S100 rabbit monoclonal antibody, ab52642, Abcam, Cambridge, UK) was performed following the same blocking procedure.

After primary antibody incubation, sections were washed several times with TBS and incubated with the appropriate biotinylated secondary antibodies for 1 h at room temperature. Signal amplification was achieved using avidin–biotin complex (ABC) detection kits, based on the high-affinity interaction between avidin and biotin. Immunoreactivity was visualized using 3,3′-diaminobenzidine (DAB) as chromogen, resulting in the formation of a colored precipitate at the antigen site.

Finally, sections were counterstained with hematoxylin for 3 min, rinsed in tap water, dehydrated through graded ethanol solutions (40-100%), cleared in 100% xylene, and mounted using Bio Mount medium (Bio-Optica, Milano, Italy) with a coverslip. Brightfield images of the stained spheroid sections were acquired using an optical microscope at multiple magnifications.

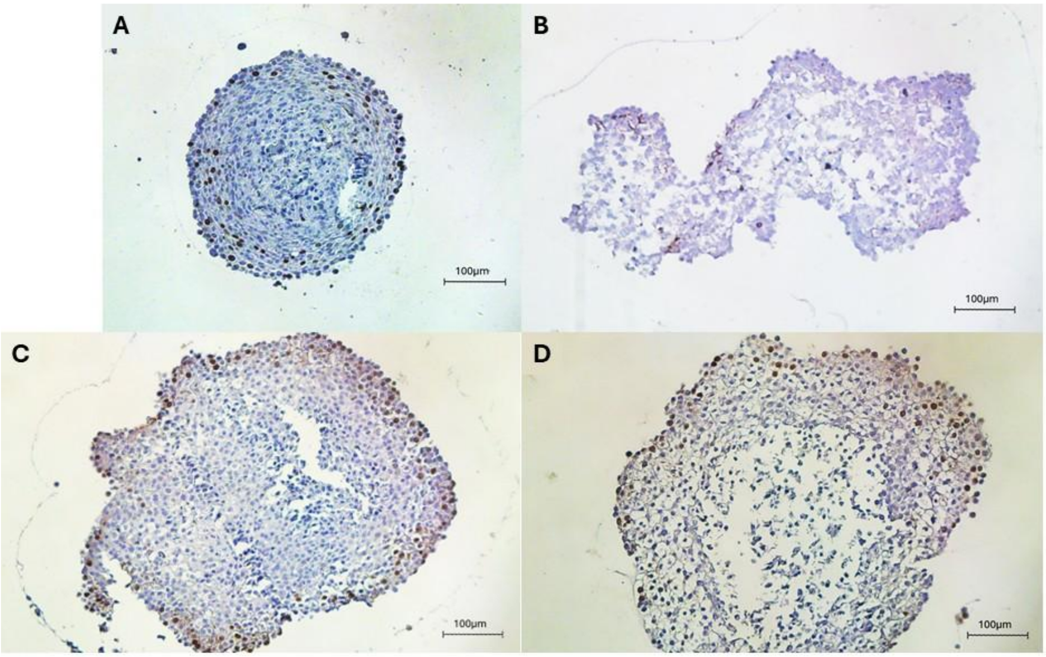

Figure S10. Immunocytochemical characterization of multicellular 3D melanoma models; transverse sections of spheroids obtained from a) 5000 L929 cells, b) 5000 B16 cells; c) 5000 L929 cells and 2500 B16 cells and, d) 2500 L929 cells and 5000 B16 cells. Immunostaining of bromodeoxyuridine-containing proliferating cells in transverse sections of multicellular spheroids: Blue (hematoxylin), black (bromodeoxyuridine).

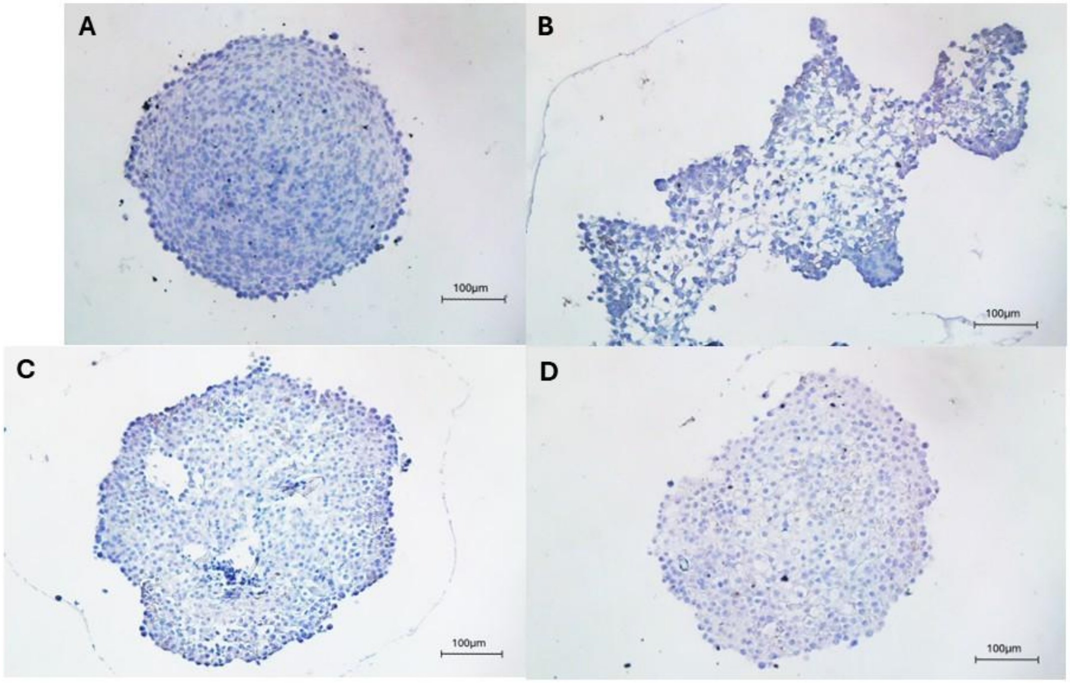

Figure S11. Immunocytochemical characterization of multicellular 3D melanoma models; transverse sections of spheroids obtained from a) 5000 L929 cells, b) 5000 B16 cells; c) 5000 L929 cells and 2500 B16 cells and, d) 2500 L929 cells and 5000 B16 cells. Immunostaining of pimonidazole-containing hypoxic cells in transverse sections of multicellular spheroids: Blue (hematoxylin), black (pimonidazole).

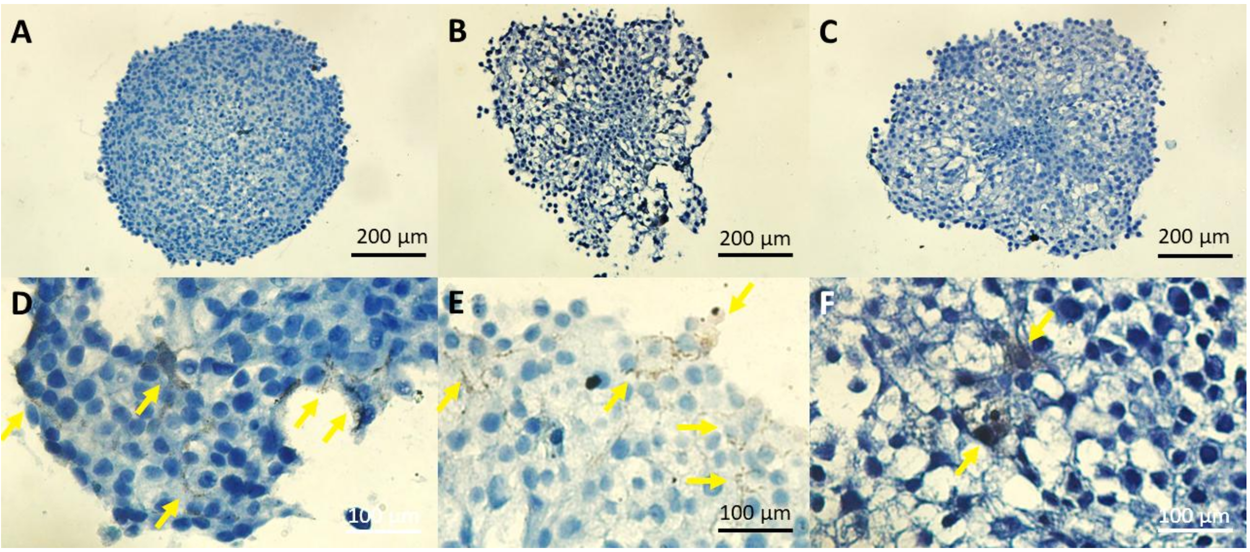

Figure S12. Immunocytochemical characterization of multicellular 3D melanoma models; transverse sections of spheroids obtained from a) 5000 L929 cells, b) 2500 B16 cells and 5000 L929 cells, and c) 5000 B16 cells and 2500 L929 cells. Immunostaining of melanoma-associated proteins in transverse sections of multicellular spheroids: d, e) Melan-A protein and f) S100 protein. Blue (hematoxylin), black (Melan-A/S100).

The 3D melanoma cell models were compositionally characterized using immunocytochemical techniques (Figures S10-S12). Cross-sectional analysis of the multicellular spheroids revealed a high degree of heterogeneity, with compact regions exhibiting morphology similar to that of L929 spheroids, mainly located in the central area of the spheroid, but not exclusively. The remaining regions displayed a loose structure, with several areas toward the center morphologically identified as necrotic in the B16-containing spheroids. All models exhibited proliferative cells positive for BrdU staining, predominantly located at the periphery of the spheroids (Figure S10). No Pimo-positive areas, indicative of hypoxia, were detected, likely due to the relatively loose spheroid morphology or the low concentration of pimonidazole used (Figure S11). Specific immunostaining confirmed the presence of proteins characteristic of malignant melanoma, namely S100 (Figure S12f, yellow arrows) and the melanoma-associated antigen Melan-A (Figure S12d,e, yellow arrows).

S5 In vivo evaluation of systemic tolerance and tissue response The study was conducted on BALB/c mice obtained from an authorized breeder. Animals were housed under standard laboratory conditions and fed a granulated commercial diet formulated for mice and rats. Food and water were provided *ad libitum* throughout the experimental period.

The animals were housed individually in standard cages, with bedding changed every three to four days. Environmental conditions were maintained at a temperature of 22-26 °C and a relative humidity of 40–60%.

All experimental procedures were approved by the Institutional Animal Ethics Committee (No. 113-CECP/8.09.2023) and conducted in accordance with EU Directive 2010/63/EU for animal experiments.

A total of 25 animals were included in the study and randomly divided into five homogeneous experimental groups, each consisting of five mice. All animals received the same test compound.

The route of administration was intravenous, via the lateral tail vein. Using a syringe equipped with a 30 G needle, 0.1 mL of the test solution was injected into the tail vein. The administered solution consisted of nanoparticles dispersed in physiological saline/ only physiological saline. Administration was performed on the same day for all animals, followed by continuous monitoring of their general health status and behavior.

The animals were euthanized at different time points following administration in order to evaluate the temporal effects of the administered nanoparticles. The first group was sacrificed at 24 hours post-administration, the second group at 48 hours, the third group at 72 hours, and the final group on day 7. Euthanasia was performed by overdose of anesthetic agents, in accordance with ethical guidelines. A complete necropsy examination was subsequently carried out, and tissue samples were collected for histological analysis.

Following necropsy, tissue samples from the spleen, liver, kidneys, lungs, myocardium, intestine, and brain were collected for histological examination. The samples were immediately fixed in 10% formaldehyde in neutral buffered for 24-48 hours at room temperature. After fixation, tissues were processed using standard dehydration protocols through graded ethanol series, cleared in xylene, and embedded in paraffin (according to the protocol described in Supplementary Materials section 3.3).

Paraffin-embedded tissue blocks were sectioned at a thickness of 4-5 µm using a rotary microtome. The sections were mounted on glass slides and initially stained with Prussian blue in order to detect ferric iron deposits and to evaluate the distribution of iron-containing nanoparticles within the examined tissues. Subsequently, the sections were counterstained with hematoxylin and eosin (H&E) for general histological assessment, including evaluation of tissue architecture and cellular morphology.

Histological examination was performed using an Olympus BX51 optical microscope, and representative microphotographs were captured using an Olympus DP74 digital camera.

Images were analyzed qualitatively for the presence of lesions, inflammatory infiltrates, necrosis, or abnormal iron accumulation.

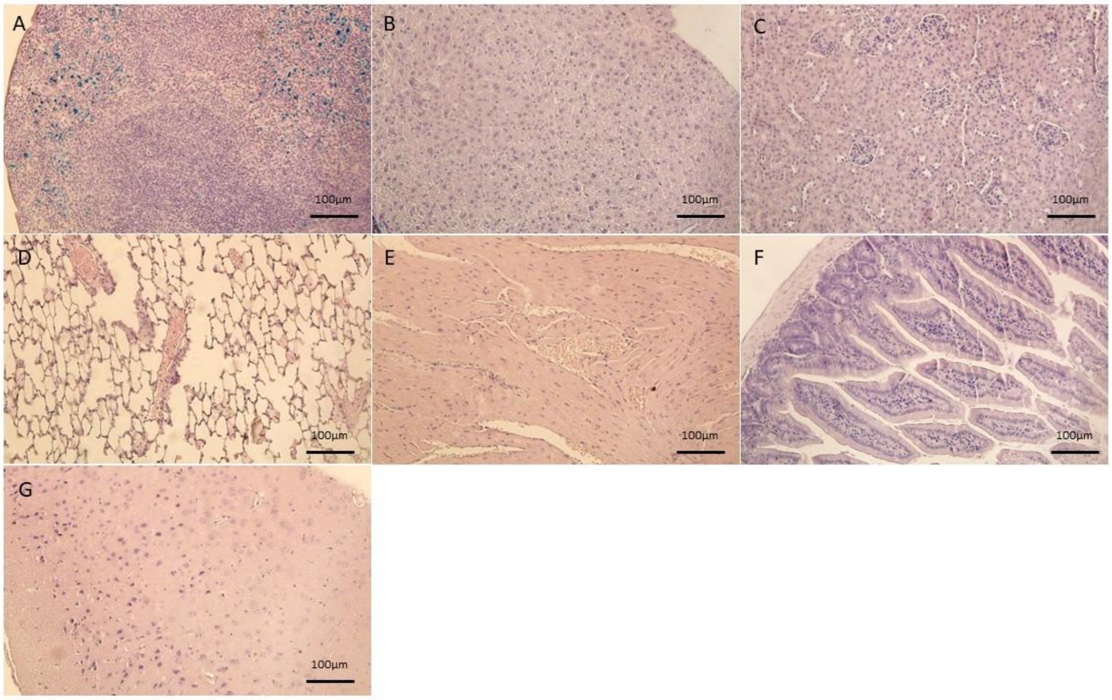

Figure S13. Histological examination of samples harvested at 24h post administration of 25%Gd-doped iron oxide nanoparticles. Cross-sections in (A) spleen, (B) liver, (C) kidney, (D) lungs, (E) myocardium, (F) intestine, (G) brain. Hematoxylin-Eosin and Prussian blue staining.

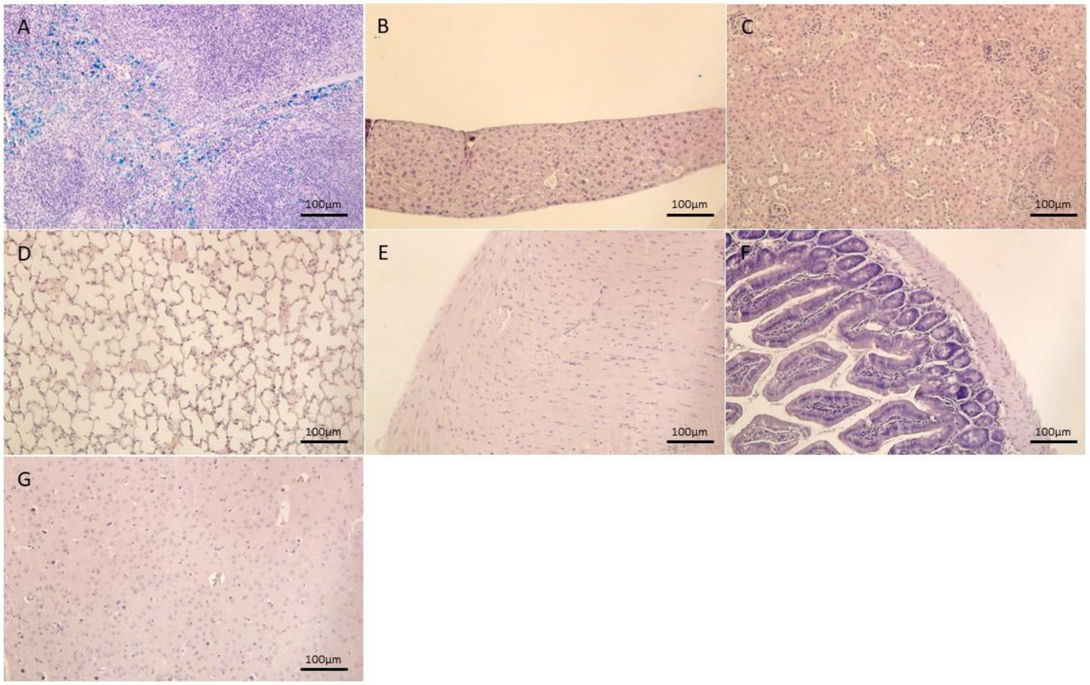

Figure S14. Histological examination of samples harvested at 48h post administration of 25%Gd-doped iron oxide nanoparticles. Cross-sections in (A) spleen, (B) liver, (C) kidney, (D) lungs, (E) myocardium, (F) intestine, (G) brain. Hematoxylin-Eosin and Prussian blue staining.

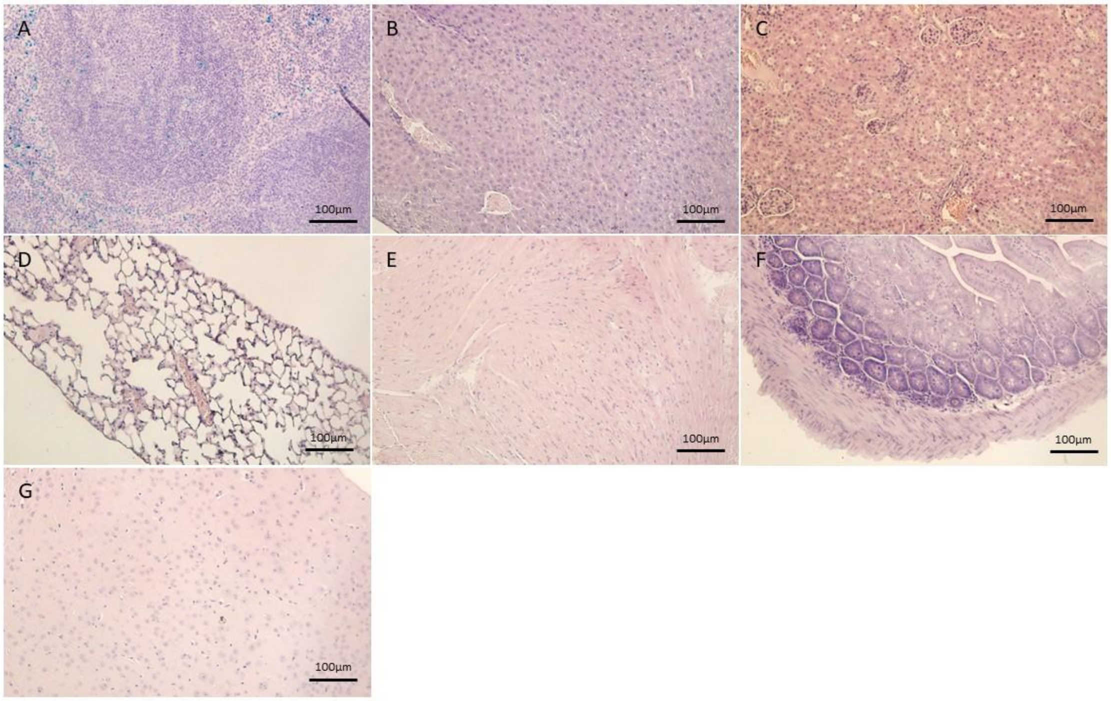

Figure S15. Histological examination of samples harvested at 72h post administration of 25%Gd-doped iron oxide nanoparticles. Cross-sections in (A) spleen, (B) liver, (C) kidney, (D) lungs, (E) myocardium, (F) intestine, (G) brain. Hematoxylin-Eosin and Prussian blue staining.

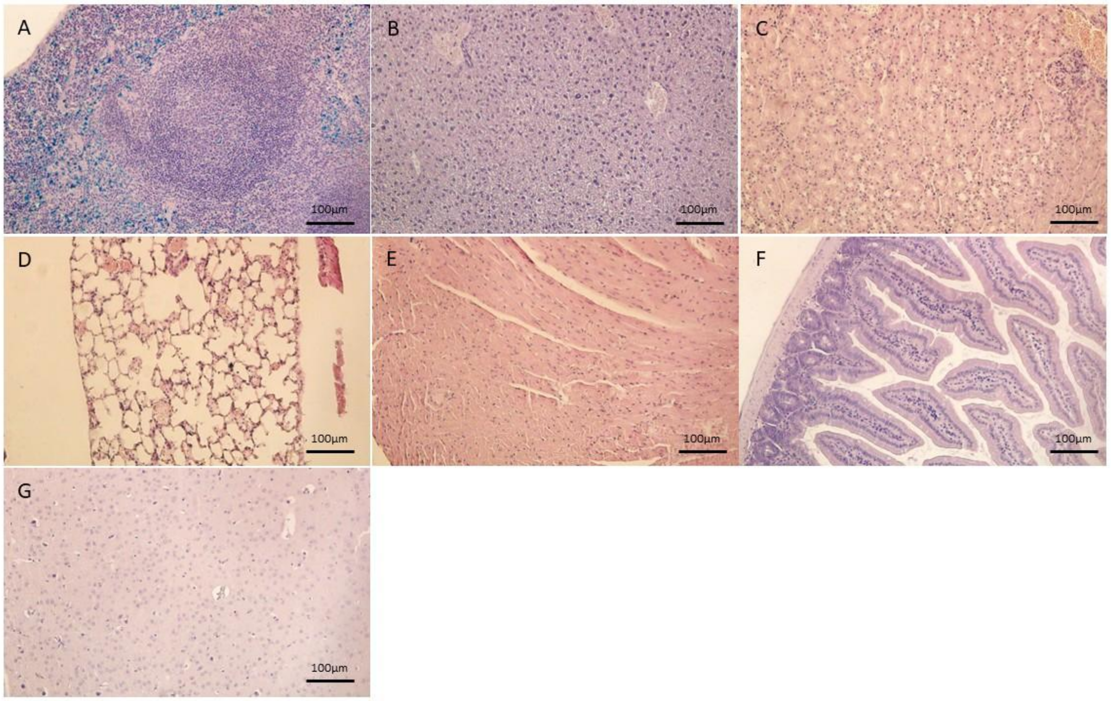

Figure S16. Histological examination of samples harvested at 7 days post administration of 25%Gd- doped iron oxide nanoparticles. Cross-sections in (A) spleen, (B) liver, (C) kidney, (D) lungs, (E) myocardium, (F) intestine, (G) brain. Hematoxylin-Eosin and Prussian blue staining.

Throughout the experimental period, all animals were closely monitored for clinical signs of toxicity or distress. Parameters assessed included general appearance, posture, grooming behavior, locomotor activity, food and water intake, and body weight evolution. No abnormal clinical signs such as lethargy, piloerection, tremors, respiratory distress, or altered behavior were observed in any of the experimental groups.

Body weight remained stable in all animals, with no statistically significant differences recorded between the groups during the monitoring period. Food and water consumption were within normal ranges. No mortality or morbidity occurred following intravenous administration of the nanoparticle formulation.

During the necropsy examination, no macroscopic lesions were observed in any of the examined organs.

A broad range of tissues was evaluated in order to assess potential damage, lesions, or inflammatory responses associated with nanoparticle administration. The organs examined included spleen, liver, kidneys, lungs, myocardium, intestine, and brain. Microscopic histological analysis did not reveal any pathological alterations or lesions in the selected organs.

Images were analyzed qualitatively for the presence of lesions, inflammatory infiltrates, necrosis, or abnormal iron accumulation.
